# Living on the edge: reconstructing the genetic history of the Finnish wolf population

**DOI:** 10.1186/1471-2148-14-64

**Published:** 2014-03-28

**Authors:** Eeva Jansson, Jenni Harmoinen, Minna Ruokonen, Jouni Aspi

**Affiliations:** 1 Department of Biology, University of Oulu, P.O. Box 3000, FIN-90014 Oulu, Finland

**Keywords:** *Canis lupus*, Museum samples, Bottleneck, Genetic drift, Effective population size, Temporal genetic data

## Abstract

**Background:**

Many western European carnivore populations became almost or completely eradicated during the last ~200 years, but are now recovering. Extirpation of wolves started in Finland in the 19th century, and for more than 150 years the population size of wolves has remained small. To investigate historical patterns of genetic variation, we extracted DNA from 114 wolf samples collected in zoological museums over the last ~150 years. Fifteen microsatellite loci were used to look at genotypic variation in this historical sample. Additionally, we amplified a 430 bp sequence of mtDNA control region from the same samples. Contemporary wolf samples (*N* = 298) obtained after the population recovery in the mid-1990s, were used as a reference.

**Results:**

Our analyses of mtDNA revealed reduced variation in the mtDNA control region through the loss of historical haplotypes observed prior to wolf declines. Heterozygosity at autosomal microsatellite loci did not decrease significantly. However, almost 20% of microsatellite alleles were unique to wolves collected before the 1960s. The genetic composition of the population changed gradually with the largest changes occurring prior to 1920. Half of the oldest historical samples formed a distinguishable genetic cluster not detected in the modern-day Finnish or Russian samples, and might therefore represent northern genetic variation lost from today’s gene pool. Point estimates of *N*_*e*_ were small (13.2 and 20.5) suggesting population fragmentation. Evidence of a genetic population bottleneck was also detected.

**Conclusions:**

Our genetic analyses confirm changes in the genetic composition of the Finnish wolf population through time, despite the geographic interconnectivity to a much larger population in Russia. Our results emphasize the need for restoration of the historical connectivity between the present wolf populations to secure long-term viability. This might be challenging, however, because the management policies between Western and Eastern Europe often differ greatly. Additionally, wolf conservation is still a rather controversial issue, and anthropogenic pressure towards wolves remains strong.

## Background

The direct and indirect influences of human activities has caused drastic changes in the genetic composition of many wild populations. Decline and fragmentation of formerly continuous populations into isolated local populations raise several genetic concerns, such as the loss of genetic variation and its associated effects on local effective population sizes (*N*_*e*_). Increase of inbreeding in small populations can ultimately lead to lower evolutionary potential and elevated extinction risk [1,2]. Small population size may also increase the risk of hybridization when the probability of finding a mate of the same species is limited [1]. Populations of highly mobile species are expected to be less prone to negative genetic effects because individuals can disperse and exchange genes across large geographic areas. Wolves are known for their great adaptability [3] and high dispersal capability (over thousand kilometres, e.g. [4,5]). Consequently, gene flow and genetic similarity between adjacent wolf populations could be expected, and large neighbouring populations may provide a buffer against loss of variation in smaller populations [6].

The grey wolf (*Canis lupus*) is one of the most controversial animals, and has been an object of intense eradication campaigns throughout Western Europe [7] and North America [8] since the 18th century. By the end of the 19th century, most of the Western European wolf populations were extirpated [9], or at least driven into isolated and fragmented habitat patches [7,10]. Also in Eastern Europe, rather extensive predator removal programmes were implemented from the 1800s onwards with a negative impact on the wolf population [7,10-12]. After WWII, alterations in forestry practices have also had large effects on wolf population sizes via their impact on the abundance of the most important prey species of wolves, moose [13,14]. Genetic studies reveal recent fragmentation in the Eastern European wolf population resulting in relatively small (local) effective population sizes [10], significant genetic differentiation and low migration rates between regions [13,15]. The historical effects of fragmentation on reductions in population size and loss of genetic variation is currently unknown.

**Figure 1:**
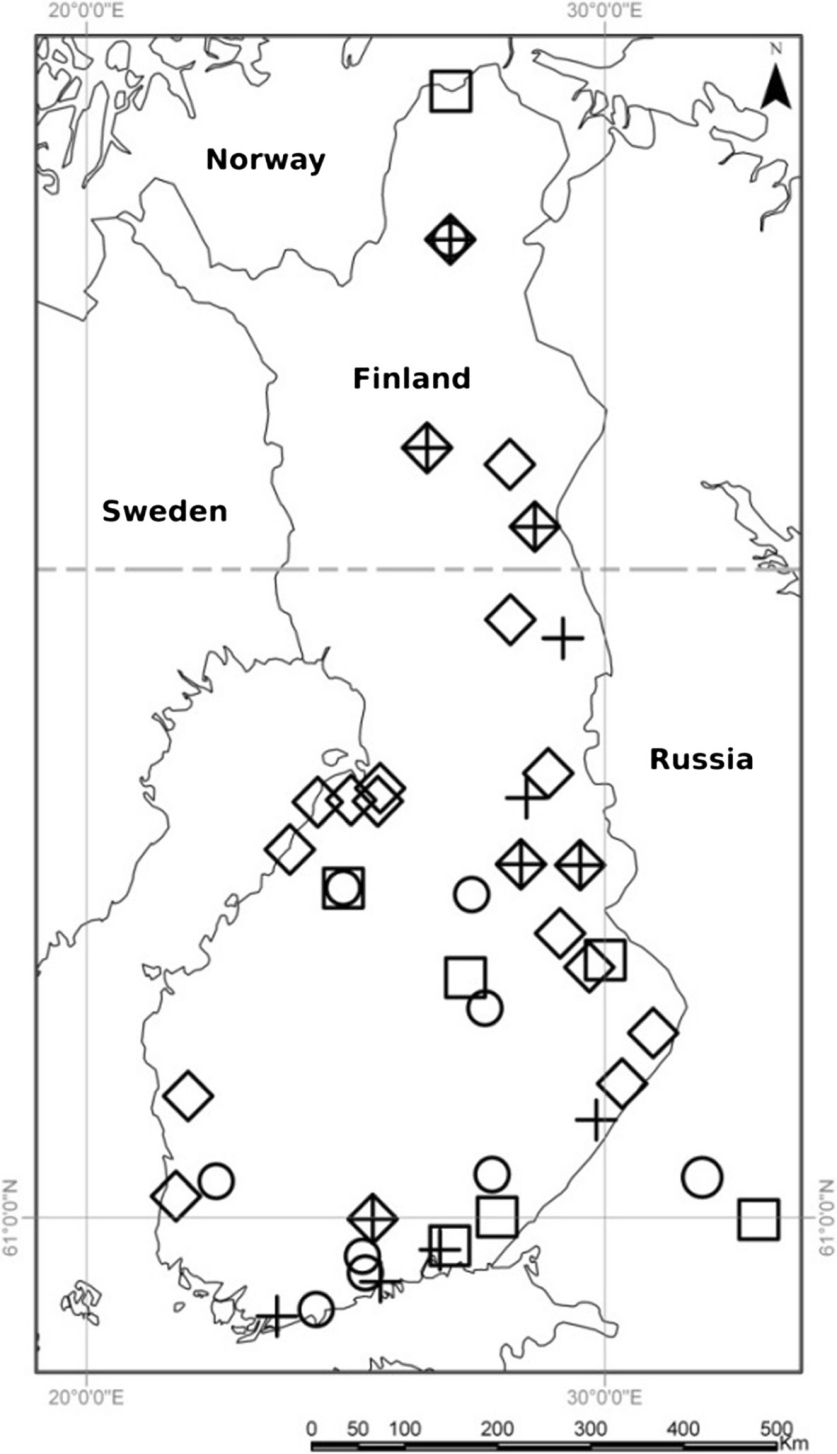
**Number of recorded wolf kills in Finland in 1845–2010 (redrawn from **[16]**, data for 1991–2010 acquired from the Finnish Game and Fisheries Institute).** Note that the time frame in the first bar (1845–1850) is shorter than in later time periods.

It has been estimated that in Finland alone, 23 000 wolves were killed during the last ~150 years ([16]; Figure 1). Before the active persecution started around the 1850s, the wolf was distributed throughout the whole Finland. Due to intense hunting wolves quickly disappeared from the western and central parts of the country [12,16]. Located on the fringe of a large Russian wolf population (currently ~40,000 wolves; [10]), the Finnish population did not presumably disappear altogether, but experienced several consecutive bottlenecks. For example, in the 1920s and 1970s, the wolf population consisted of a few individuals [12,16,17]. It has been suggested that the whole population was extirpated in the 1920s because of a distemper epidemic [18]. In 1973, the wolf became protected in Finland outside the northern reindeer management area. Until the 1990s, the population size was mostly regulated by the amount of incoming immigration from the source population in north-western Russia [13,19]. However, when wolves started to regularly reproduce in Finland after the middle 1990s, the Finnish population became somewhat genetically differentiated from the Russian one [13,20], and the Finnish population numbers did not any more reflect the abundance variation of the Russian population [19].

The use of museum collections in population genetics studies may provide a valuable historical perspective for the conservation and management of present-day populations. Specimens in museum collections often provide information on genetic diversity prior to population declines in response to anthropogenic effects [21], and therefore provide a baseline against which to evaluate the current genetic status of the species. Historical samples have been successfully used in many genetic studies since the early 1990s (for reviews see [22-24]), commonly investigating the genetic consequences of different anthropogenic factors, such as overexploitation [25-27], habitat fragmentation [28-30] or even direct persecution [6] on the genetic diversity and structure of populations.

In the present study, we used DNA extracted from wolf samples obtained from zoological museums to examine historical patterns of genetic variability and structure in the Finnish wolf population, which experienced a dramatic population decline over a ~150 year period. We were further interested to see the possible stabilizing effect of gene flow from the neighbouring, large eastern population, i.e. could gene flow from Russian population have been large enough to prevent negative consequences (e.g. the loss of genetic variation and small *N*_*e*_) associated with population decline.

## Results

### DNA extraction and amplification

**Figure 2:**
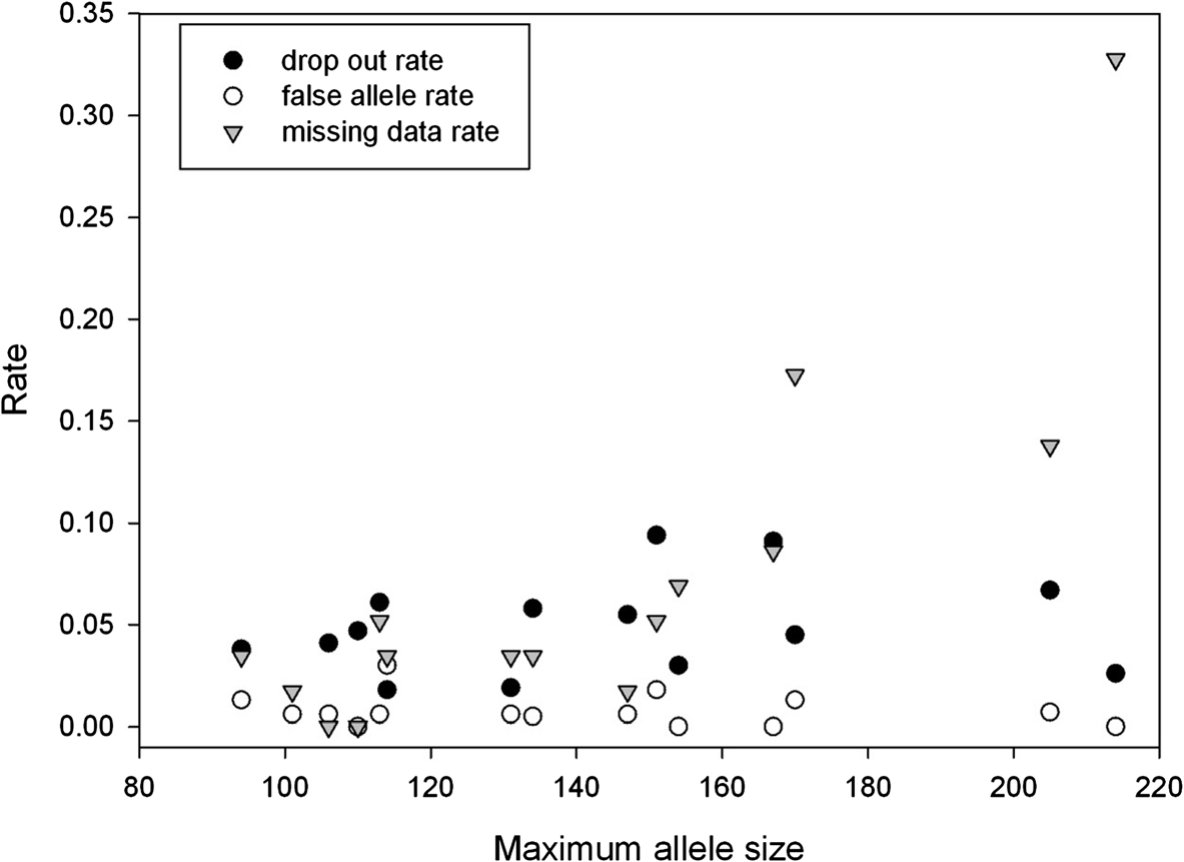
**Geographic location of samples used.** Samples collected prior to 1920 are shown with circles, 1920–1959 with squares, 1960–1979 with diamonds and 1980–1993 with crosses. Two samples located outside Finland were inside the country borders at the sampling date.

A total of 114 samples (Figure 2; Additional file 1: Table S1) were extracted and 89 (78.1%) were successfully amplified with mitochondrial primers and 66 (57.9%) with at least 8 of 15 microsatellite loci. Amplification success varied between the temporal groups and sample types (Additional file 2: Table S2). In agreement with previous studies, the success rate was higher for mtDNA than for microsatellites, bone samples amplified on average better than the pelt samples [31], and tissue samples from dental cavities were a very good source of usable DNA [6].

**Figure 3:**
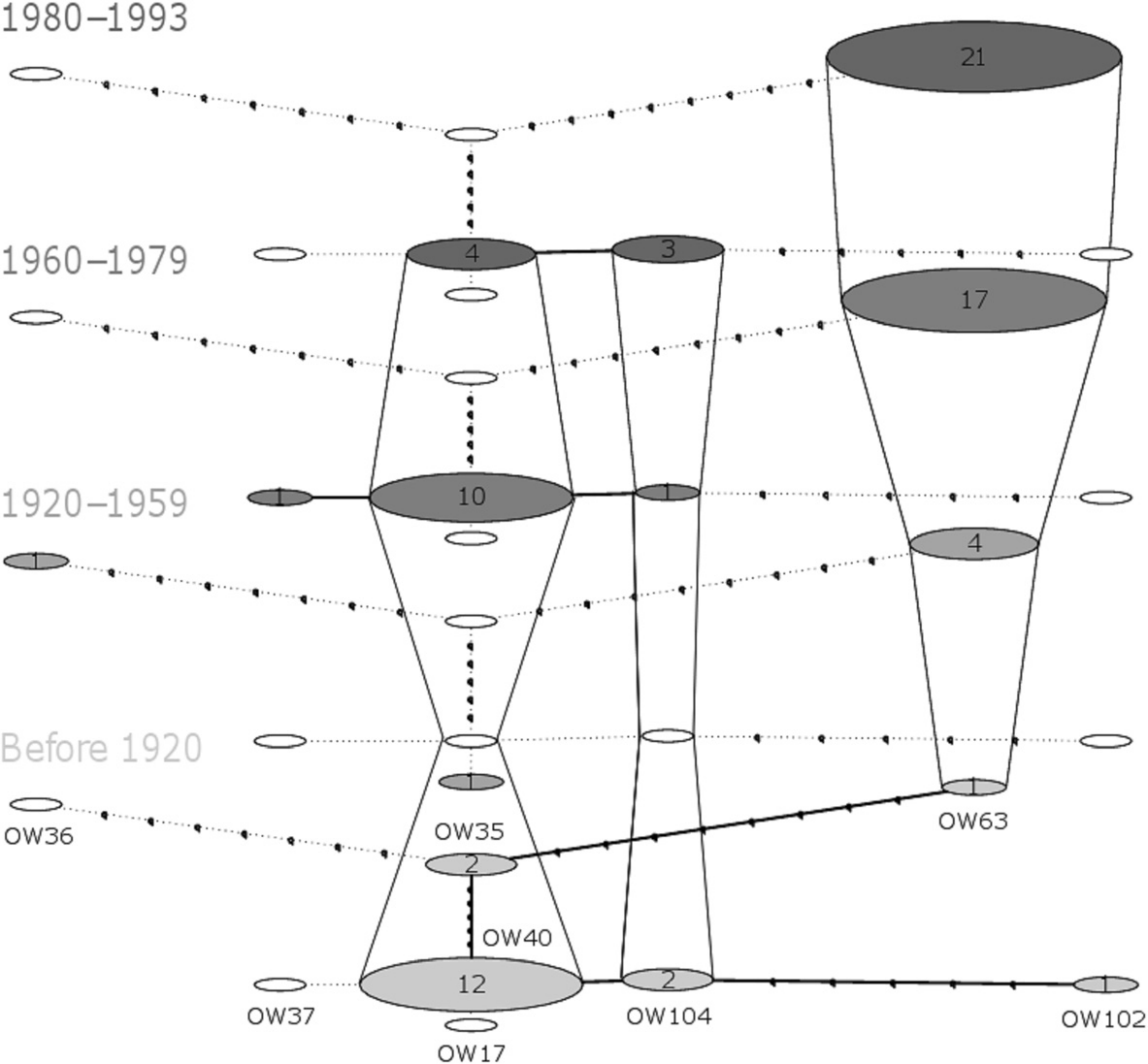
**Amplification success and detected error rates compared to maximum allele sizes in used 15 microsatellite loci.** Amplicon length had a significant effect on amplification success (correlation between missing data rate and maximum allele size in locus: *r* = 0.828, *P* < 0.001), but not on detected error rates (*P* > 0.05).

We did not observe a significant correlation between the microsatellite amplicon length (measured as maximum allele size) and error rates (the number of drop outs and false alleles; Figure 3). On the other hand, amplification success was greatly reduced on loci with long allele sizes (~170 bp or larger; “missing data rate” in Figure 3). MICROCHECKER [32] analysis did not suggest any scoring errors due to stuttering or large allelic drop outs in any of the temporal groups. Null alleles were possibly present in locus CPH12 in the population sampled in 1980–1993 with 13 observed homozygotes compared to expected 10.6. Because null alleles at this locus were not detected in the other temporal groups, the locus was included in further analyses.

Ten samples were discarded from the data set prior to genetic analyses. Of these ten, eight were discarded due to close relatedness (i.e. pseudo-replicates were removed; [33]) indicated by their genotypes. Two samples had identical genotypes and collection information, and one of them was discarded. Additionally, one mitochondrial sequence was not included in further analysis due to a repeatedly amplified double peak in two nucleotide positions, possibly due to post-mortem C → T deamination [34,35]. In the first temporal group (samples collected prior to 1920) 18 mtDNA sequences and 12 microsatellite genotypes were analysed, in the second (1920 – 1959) 6 of both types, in the third (1960 – 1979) 29 and 22, and in the fourth (1980 – 1993) 28 and 18.

### Mitochondrial sequence analyses

#### ***mtDNA variation and genetic differentiation between temporal groups***

**Figure 4:**
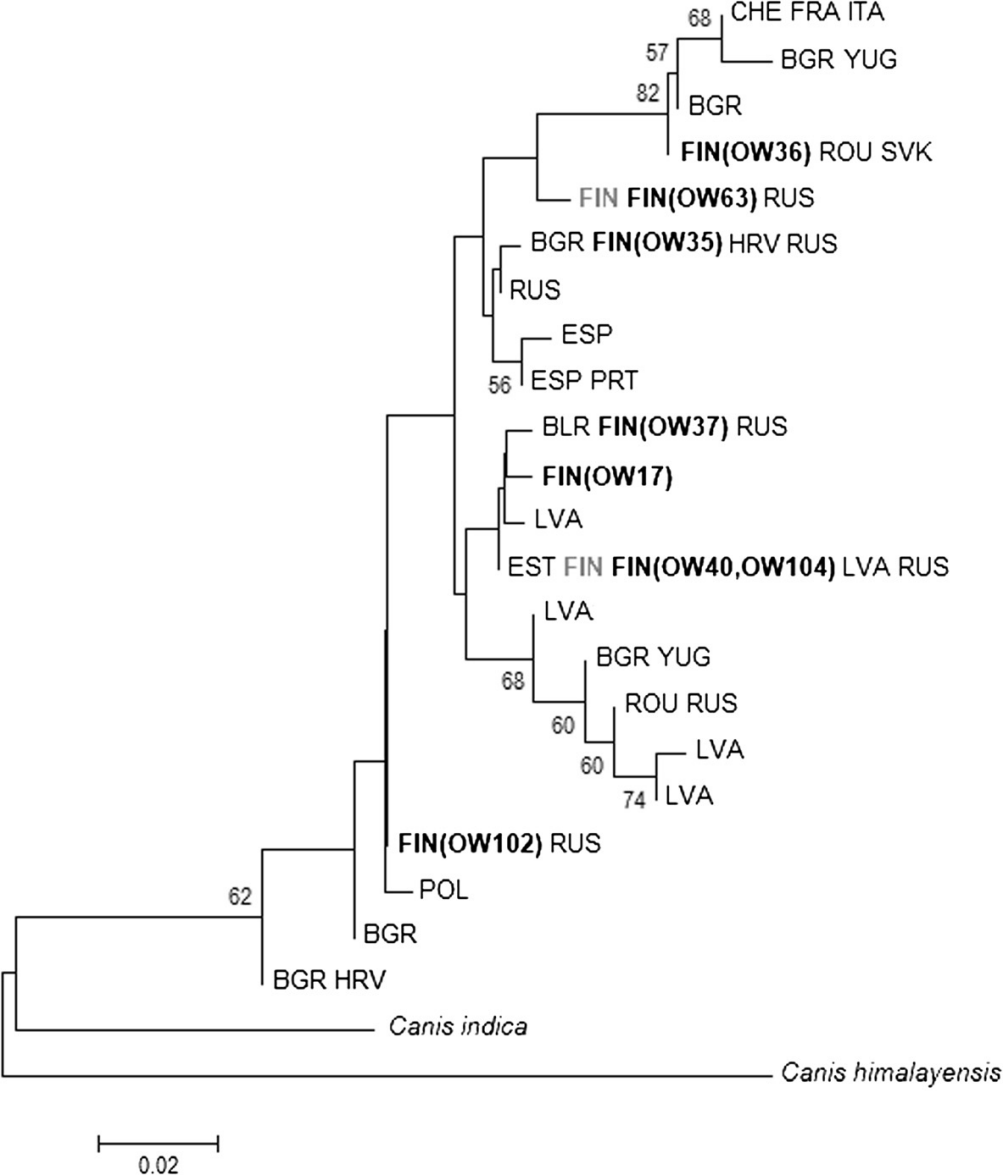
**Temporal haplotype network for historical Finnish wolves.** Each circle represents a different haplotype whose names are given next to the circles in the bottommost layer. Number of individuals bearing each haplotype is given within circles. White circles denote missing haplotypes for that time period. Number of dots +1 connecting haplotypes equals to nucleotide differences. Maximum evolutionary divergence (i.e. base substitutions per site) was 0.055 (OW36–OW102) and minimum 0.003 (OW40–OW17/OW37/OW104).

A total of 23 nucleotide positions were found to be polymorphic within the amplified 431 bp long mtDNA sequences yielding eight distinct haplotypes (Figure 4). The three most common haplotypes (OW63, OW40 and OW104) in historical wolves were the ones present also in the current wolf population [36]. These haplotypes were present in 43, 26 and 6 historical wolves (respectively). The remaining five haplotypes were rare and found only in one (OW17, OW36, OW37 and OW102) or two (OW35) samples.

Mitochondrial diversity measured as number of haplotypes and number of polymorphic sites was highest (Table 1) at the beginning of the sampling period (until 1959) despite smaller sample sizes. Other haplotype diversity estimates followed the same pattern and were clearly or slightly higher in samples collected before 1960 (see Table 1 for details). Genetic differentiation based on haplotype frequencies (Φ_*ST*_) was large (0.329–0.514) and highly significant (*P <* 0.001) between the first (before 1920) and all other temporal groups (Table 2), but not in other pairwise comparisons. Three main haplotypes (OW40, OW63 and OW104; Figure 4) were found in all except the small second group (1920–1959, *N* = 6), in which four individuals carried the haplotype OW63 and other two had unique haplotypes (OW17 and OW36). The large difference between the first and other groups was mostly due to the shift between the two dominating haplotypes; in the first group 12 of the 18 individuals (66.6%) had the OW40 haplotype and only one OW63 (5.6%), whereas among wolves collected after 1960 abundances were reversed. In 1960–1979 ten individuals of 29 (34.5%) had haplotype OW40 and 17 (56.6%) OW63, and in 1980–1993 the proportions (respectively) were 4/28 (14.3%) and 21/28 (75.0%).

**Table 1:** Mitochondrial diversity and neutrality tests for temporal Finnish wolf groups

**Temporal sample**	***N***	***H***	***H***_***R***_	***P***_***R***_	***H***_***d***_	π	***S***	***D *(*P *)**	***F***_***S ***_**(*P *)**
Before 1920	18	5	2.80	1.13	0.556 ± 0.130	0.0070	15	-1.17 (0.89)	1.71 (0.18)
1920-1959	6	3	3.00	2.00	0.600 ± 0.215	0.0132	14	-0.47 (0.61)	3.27 (0.06)
1960-1979	29	4	2.35	0.25	0.554 ± 0.064	0.0108	11	2.15 (< 0.01**)	6.62 (0.01*)
1980-1993	28	3	2.17	0.18	0.421 ± 0.103	0.0086	10	1.41 (0.06)	7.02 (0.01*)

*N* sample size, *H* number of haplotypes, *H*_*R*_ haplotype richness, *P*_*R*_ private haplotype richness, *H*_*d*_ haplotype diversity, π nucleotide diversity, *S* number of polymorphic sites, *D* Tajima’s D and *FS* Fu’s FS with corresponding *P* value; *< 0.05, **< 0.01.

**Table 2:** Genetic differentiation among temporal Finnish wolf groups

	**Before 1920**	**1920-1959**	**1960-1979**	**1980-1993**	**1995-2009**
Before 1920	–	0.487***	0.329***	0.514***	NA
1920-1959	0.052*	–	-0.014 NS	-0.050 NS	NA
1960-1979	0.096***	0.038*	–	0.023 NS	NA
1980-1993	0.066***	0.061***	0.033***	–	NA
1995-2009	0.089***	0.041***	0.024***	0.022***	–

In upper right corner Φ_*ST*_- values for mtDNA, in lower left corner *F*_*ST*_ - values for microsatellite markers.

*P* - values: ***< 0.001, *< 0.05, NS not significant, NA, data not available.

#### ***mtDNA population bottleneck tests***

Neutrality tests suggested significant demographic changes in the wolf population at the end of the historical sampling period (Table 1). The two neutrality tests were significant in temporal groups collected in 1960–1979 (for Tajima’s *D P* = 0.0013 and for Fu’s *F*_*S*_*P* = 0.011), whereas for the group collected in 1980–1993, Fu’s test value *F*_*S*_ was significant (*P* = 0.006), but Tajima’s *D* showed a decreasing trend (*P* = 0.059).

#### ***European-wide mtDNA phylogeny***

**Figure 5:**
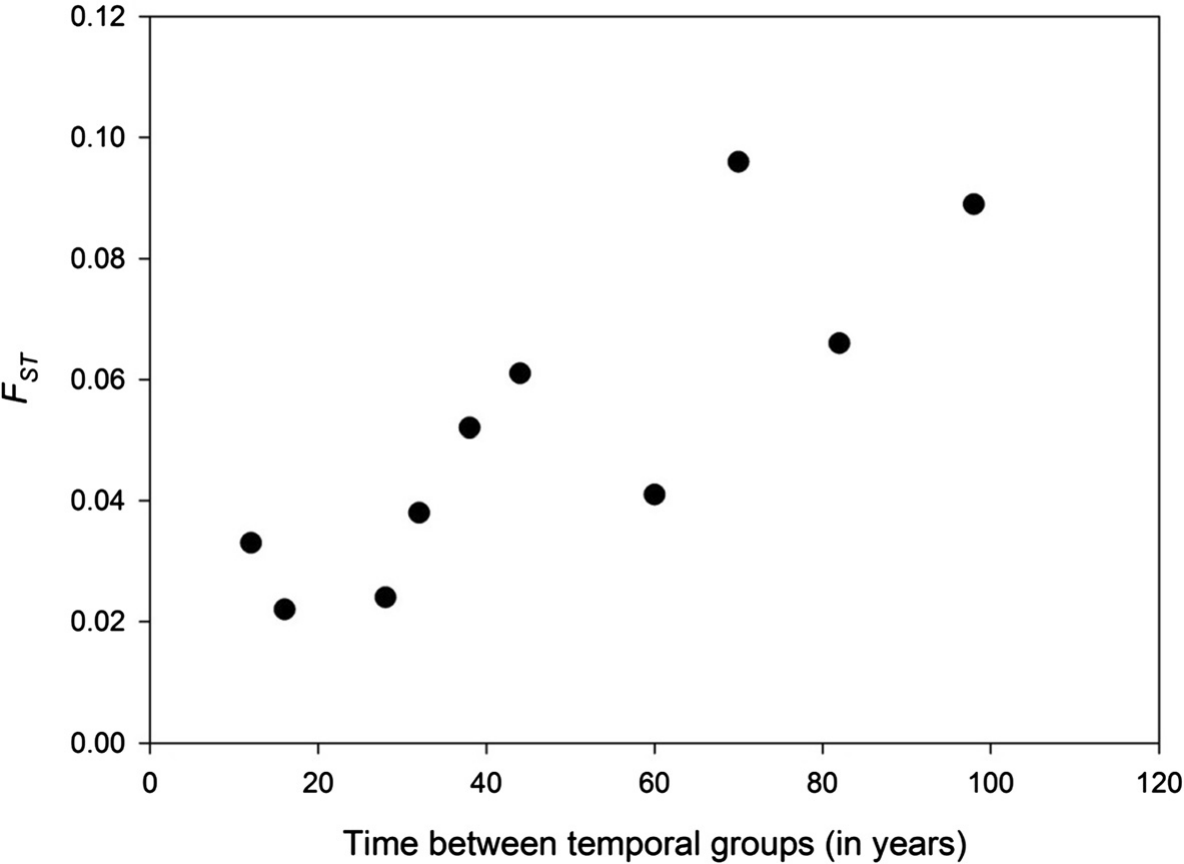
**European-wide wolf mtDNA haplotype tree (*N***_***sequences***_ **= 497; 287 bp, NJ) together with the haplotypes found in the historical Finnish wolf population FIN (black) and those still existing today FIN (grey).** Names of the sequences refer to the land or geographic area reported for them. Bootstrap values above 50 are shown.

The overall dataset including our 81 historical samples consisted of 497 sequences with the mtDNA sequence length of 287 bp. In total, 21 different haplotypes among European wolves were detected (Figure 5; phylogeny based on 390 bp sequences is given in Additional file 3: Figure S1).

Two historical wolf haplotypes that are also found in the contemporary Finnish population (OW40 and OW104; Figure 4) were merged into a single one when sequences were truncated. The remaining seven historical haplotypes comprised one that was unique and six haplotypes that have previously been reported from other parts of Europe. Interestingly, the still-existing Finnish wolf haplotypes (OW63 and OW40/104; Figure 4) have mainly been found in nearby countries (Russia and Estonia), whereas those historical haplotypes that have vanished from the modern Finnish population, seem to have a much wider geographical distribution (Figure 5).

### Microsatellite analyses

#### ***HW-equilibrium and linkage disequilibrium between loci***

The oldest (prior to 1920) and the latest (1980–1993) temporal groups deviated significantly from HW-equilibrium with several loci showing heterozygosity deficiency (global multilocus tests *P* = 0.016 and 0.002, respectively), whereas the historical temporal groups 1920–1959, 1960–1979, and the modern reference sample (1995–2009) were in HW-equilibrium. None of the 15 loci showed a constant pattern of deviation (indicating e.g. amplification errors). Significant linkage disequilibrium (*P <* 0.001) between loci was detected in 7 of the 105 pairwise comparisons in samples collected prior to 1920, 2/105 in 1920–1959, 2/105 in 1960–1979, 3/105 in 1980–1993 and 1/105 in 1995–2009.

#### ***Amount of genetic variation and inbreeding coefficient***

We found no significant differences in the amount of expected or observed heterozygosity among the temporal groups, and there was no evidence of inbreeding (Table 3; see Additional file 4: Table S3 for locus specific results and for *F*_*IS*_ results). Allelic richness estimates were also rather similar throughout the study period, whereas private allelic richness was clearly higher in samples collected prior to 1960 (0.59 and 0.45 vs. 0.22–0.27; Table 3). When we compared diversity indices (*H*_*o*_, *H*_*e*_ and *A*_*R*_) of samples collected before and after 1960 using the randomization test, no notable differences between the groups were found except for a slightly higher (but not significant; *P* = 0.092) allelic richness in the older group (3.947 vs 3.655).

**Table 3:** **Microsatellite diversity and *N***_***e ***_**point estimates for temporal Finnish wolf groups**

**Temporal sample**	***N***	***He *(σ)**	***Ho *(σ)**	***A***	***A***_***R***_	***P***_***R***_	***LD-N***_***e ***_**(95% CIs)**	***N***_***e ***_***by ONeSAMP *(95% CIs)**
Before 1920	12	0.669 (0.131)	0.624 (0.148)	5.00	3.72	0.59	20.5 (13.5 - 35.2)	13.2 (10.8 - 19.6)
1920 - 1959	6	0.721 (0.061)	0.741 (0.238)	4.73	4.18	0.45	*NA*	*NA*
1960 - 1979	22	0.686 (0.088)	0.729 (0.113)	5.47	3.60	0.22	76.4 (48.1 - 159.0)	24.3 (20.9 - 32.7)
1980 - 1993	18	0.676 (0.139)	0.622 (0.178)	5.53	3.70	0.27	45.2 (30.4 - 78.4)	23.1 (18.5 - 36.3)
1995 - 2009	30	0.697 (0.067)	0.712 (0.095)	5.93	3.67	0.23	99.2 (65.1 - 176.0)	37.2 (31.2 - 58.3)

*N* sample size, *He* expected heterozygosity (standard deviation), *Ho* observed heterozygosity, *A* number of alleles, *A*_*R*_ allelic richness, *P*_*R*_ private allelic richness, LD-*N*_*e*_ effective population size estimate based on linkage disequilibrium, *NA* not analysed due to small sample size.

#### ***Ghost alleles***

One hundred and seven alleles were found among all samples with the 15 microsatellite loci used (Additional file 5: Figure S2). Twenty-one alleles (19.6%) present in historical wolves were not found from much larger modern reference sample (1995–2009, *N* = 298), whereas only four (3.7%) alleles were unique to the present wolf population. Of these 21 historical alleles, nine (42.9%) were only found from samples prior to 1960 (*N* = 18) and four (19.0%) from samples collected in 1960–1993 (*N* = 40). Because the sampling of present-day wolf population is rather comprehensive (on average 42% of the wolves born that time; [20]), it is legitimate to presume, that these alleles have vanished from the modern Finnish wolf population, and represent true ‘ghost alleles’ and that suggest a more diverse historical population. Besides high occurrence of alleles unique to museum samples, we detected large frequency shifts in many alleles (Additional file 5: Figure S2) indicating genetic drift.

#### ***Genetic differentiation***

**Figure 6:**
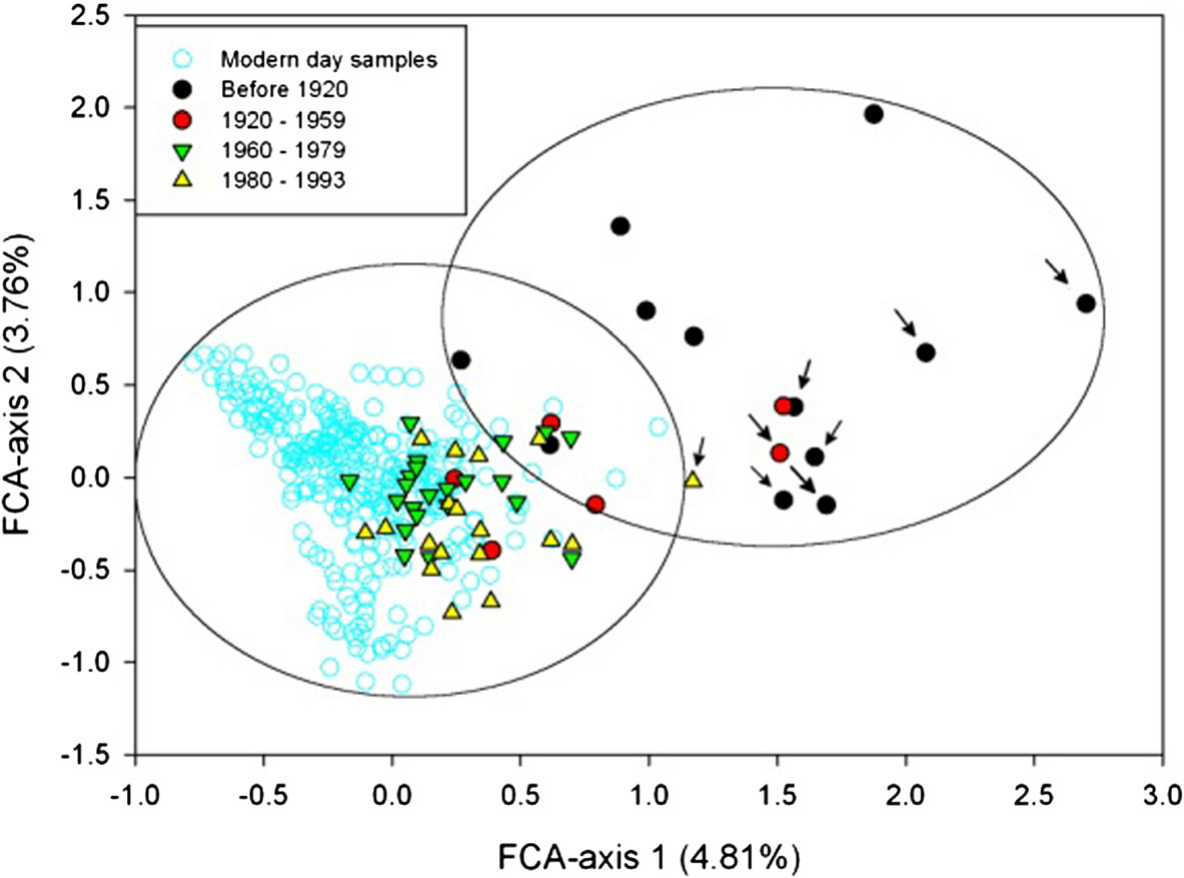
**The amount of genetic differentiation (*F***_***ST***_**) between temporal sample pairs based on microsatellite markers.** For each temporal group median year was calculated and used as a time point for the whole sample. Mantel test for correlation of matrices: *r =* 0.838, *Z*_*data*_ = 30.61, *Z*_(average from 9999 permutations)_ *=* 25.04, *p* = 0.048.

The genetic composition of the Finnish wolf population clearly changed over time. Genetic differentiation between the temporal groups was small or moderate (*F*_*ST*_ = 0.022–0.096; Table 2), but statistically significant in all cases. The pairwise genetic distances (*F*_*ST*_) between the temporal groups were significantly correlated with the elapsed time (i.e. differences in median sampling year of the groups; Mantel’s test: *r =* 0.838, *P* = 0.048; Figure 6). Relationship between the genetic and temporal distances was linear suggesting that the change in the population gene pool can be described as a gradual change. According to AMOVA, ~5.2% of the total genetic variation was detected among the temporal groups and ~94.8% within the groups (*P* ≈ 0).

**Figure 7:**
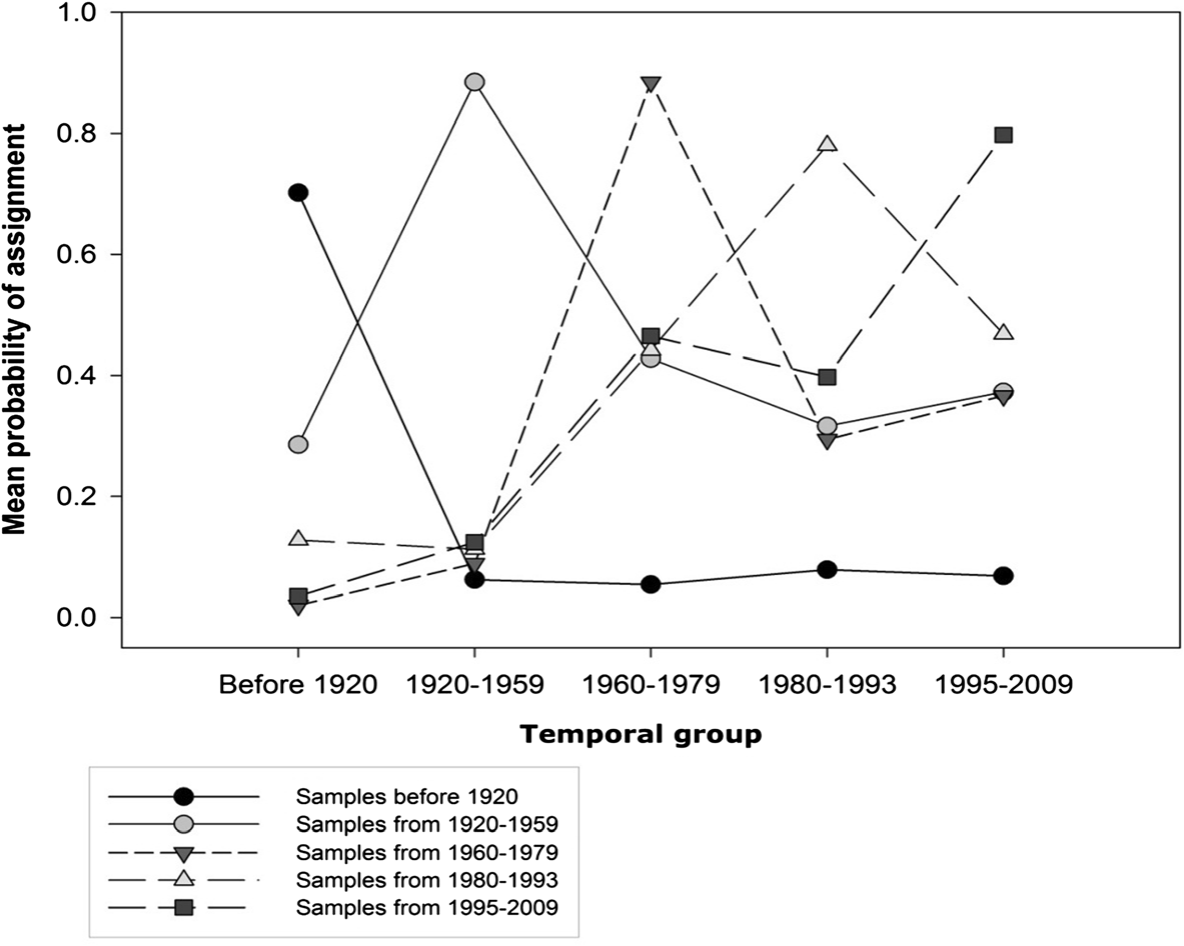
**FCA plot for the historical temporal groups together with wolf samples collected after the population recovery (*N*** **= 298 from 1995–2009; **[20]**).** The approximate distribution of oldest (collected before 1920) and modern-day groups are shown with ovals. Arrows show the northern wolves forming a distinctive cluster in Structure analysis.

The two-dimensional FCA-plot of the distribution of genetic variation between wolves (Figure 7) also suggested a gradual change in the population. Largest distance was seen between the oldest (before 1920) and modern-day wolves (1995–2009). Observed means and variances of distribution patterns differed significantly between the temporal groups along both FCA-axes indicating that historical wolves were genetically different compared to present-day wolves. Much higher standard deviations of the FCA-scores among the oldest compared to present-day samples showed that the distribution of genetic variation has been much wider historically. Statistical tests for the distribution patterns are given in Additional file 6: Text S1.

**Figure 8:**
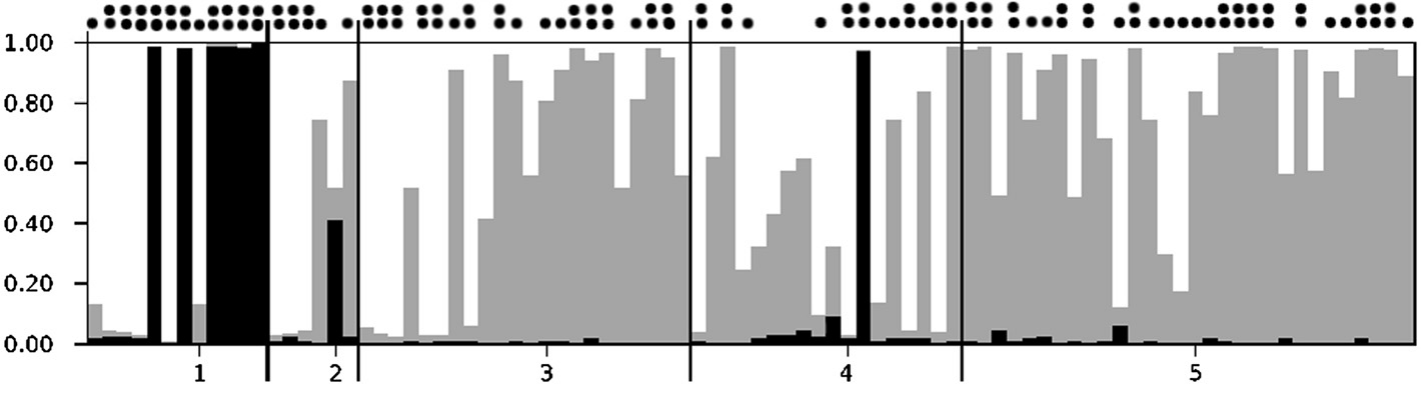
Probability assignments of the Finnish wolves into different temporal groups.

The assignment analysis showed varying degree of genetic similarity among the museum groups (Figure 8). All individuals were assigned with the highest probability to their own temporal group. Interestingly, the probabilities of sample assignments to other than their own temporal group varied greatly. Especially, the proportion of assignments into the first temporal group (prior to 1920) was low (5.4% - 7.9%) among the individuals of the subsequent groups indicating relatively large genetic changes between the first and following time periods. The wolves in the other temporal groups had also rather small mean probabilities (< 12.5%) to belong to the second temporal group suggesting rather unique composition of this group. On the other hand, mean assignment probability of wolves in the second temporal group to the latter groups were quite high suggesting that in the subsequent periods there were still substantial amount of genetic variation which was present in the second temporal group. Samples collected after 1959 showed much more admixture and the mean assignment probabilities of the wolves to other than their own or first temporal group were 0.29–0.47.

Genetic differentiation between the current Karelian wolf population (1995–2010) and the oldest temporal group of the Finnish wolves (prior to 1920) was significant, but smaller (*F*_*ST*_ = 0.047, *P* = 0.002) than any of the estimates between the oldest and other temporal Finnish groups (*F*_*ST*_ = 0.052–0.096; Table 2). This suggests that historical genetic variation might be better preserved in the neighbouring large Russian population. However, the inspection of the distribution of variation (Additional file 7: Figure S3) revealed a very similar pattern to that seen among the Finnish wolves (Figure 7). There was very little overlap between these two groups and the current Karelian distribution is more compact than the scores of the historical Finnish wolf population.

#### ***Hidden population structure***

**Figure 9:**
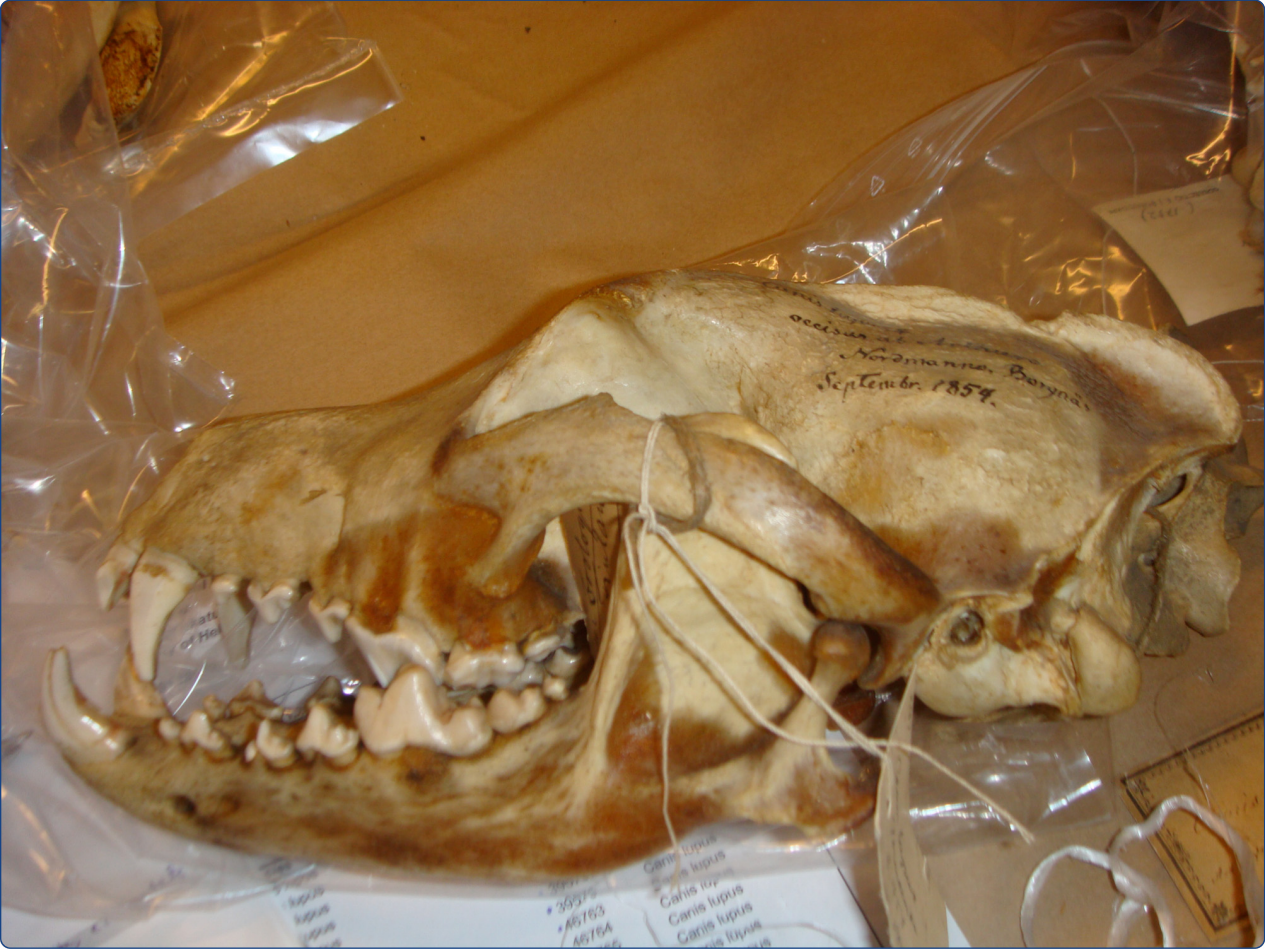
**Assignment of the Finnish wolves in each temporal group with the most likely number of genetic clusters (*K*** **= 3).** Each vertical bar represents one individual with different grayscale segments showing the likelihood of belonging to defined clusters (1 = samples collected prior to 1920, 2 = 1920–1959, 3 = 1960–1979, 4 = 1980–1993, 5 = 1995–2009). Black circles above bars show assignment levels of q = 0.7–0.9 (one circle) or q > 0.9 (two circles).

Structure analysis revealed hidden spatial and temporal population structure within the historical Finnish wolf population. According to the ad hoc quantity ∆*K *[37], the most likely number of clusters was three (Additional file 8: Figure S4). 80.7% of the individuals were inferred to these clusters with an assignment level of at least 0.7, and for over a half of samples (55.7%), the level was over 0.9 (Figure 9). The contribution of the three inferred clusters to the genetic composition in different temporal groups was highly concordant between different runs (data not shown), and indicated change in genetic structure over time. Especially the first group (prior to 1920) showed a very distinct composition compared to later time periods: half of the samples (6/12) belonged to a cluster with very little admixture with the other two clusters (q > 0.9; black bars in Figure 9), and only two wolves were inferred to this group afterwards (one admixed individual with q ~0.4 sampled in 1954 and another with an assignment of 0.97 from 1990). Interestingly, all of these wolves were sampled from Northern Finland (whereas no geographic distribution pattern for the two other clusters was detected; data not shown). Additionally, wolves belonging to another genetic subgroup (grey bars) were common in samples from 1920 onwards and most common among the modern-day reference samples (1995–2009; in 21 out of 30 with q ≥ 0.7), but were almost absent in the first temporal group.

#### ***Demographic changes***

Bottleneck tests based on the heterozygosity excess method did not indicate severe preceding bottlenecks in any of the temporal groups or in the modern-day reference sample. L-shaped allelic frequency distribution typical to non-bottlenecked populations (Additional file 9: Figure S5) was observed and one-tailed Wilcoxon tests for the heterozygosity excess gave a *P* value of > 0.05 in all except the second temporal group (1920–1959, *P* = 0.0206), which was too small for reliable testing (*N* = 6). Even though alleles frequency distribution was L-shaped in all groups, the proportion of rare alleles (frequency ≤ 0.1) was lower in the first two temporal groups (prior to 1920 and 1920–1959, *F =* 0.333 and 0.254, respectively) than in following samples (*F* = 0.390/0.386/0.472), and as a consequence, the distribution more shallow.

*M*-ratio tests revealed high average *M*-ratios across the temporal groups (0.794–0.910; Table 4). Comparison of observed ratios against simulated mutation-drift equilibrium expectations showed, however, that unless the proportion of non-single-step mutations is high (≥ ~0.3) and/or the effective size of the population large (*N*_*e*_ *≥* 500 and corresponding Θ-value 0.4 or larger), the occurrence of a genetic bottleneck in the first temporal sample was supported. Under the evolutionary scenarios investigated, bottlenecks for other temporal groups are supported only if the mutation model follows a strict step-wise mutation model (SMM) or the proportion of larger than single-step mutations is at most ~10%.

**Table 4:** ***M*-ratio simulations for the temporal groups**

**Table a) shows the proportion of simulated equilibrium populations with smaller than observed *M-*ratio**								
**a)**	**Before 1920, *N*** **= 12, *M*** **= 0.794**				**1960 - 1979, *N =*** **22, *M*** **= 0.910**			
	**Θ**				**Θ**			
***pg***	0.04	0.08	0.2	0.4	0.04	0.08	0.2	0.4
0.0	**0**	**0**	**0**	**0**	**0**	**0**	**0**	**0.002**
0.05	**0**	**0**	**0**	**0**	**0.030**	**0.039**	0.059	0.112
0.1	**0.001**	**0.002**	**0.003**	**0.005**	0.149	0.166	0.224	0.342
0.2	**0.025**	**0.035**	**0.049**	0.088	0.510	0.552	0.642	0.754
0.3	0.147	0.166	0.217	0.327	0.803	0.832	0.889	0.944
	**1980 - 1993, *N =*** **18, *M*** **= 0.856**				**1995-2009, *N*** **= 30 , *M*** **= 0.869**			
0.0	**0**	**0**	**0**	**0**	**0**	**0**	**0**	**0**
0.05	**0.002**	**0.001**	**0.004**	**0.009**	**0.003**	**0.004**	**0.008**	**0.016**
0.1	**0.021**	**0.025**	**0.040**	0.071	**0.032**	**0.037**	0.064	0.104
0.2	0.182	0.199	0.264	0.380	0.243	0.255	0.331	0.468
0.3	0.487	0.587	0.597	0.719	0.561	0.594	0.688	0.782
**Table b) shows the corresponding *Mc *-values (limit for 5% of simulations with lowest *M*-ratio)**								
**b)**	**Before 1920, *N*** **= 12, *M*** **= 0.794**				**1960 - 1979, *N =*** **22, *M*** **= 0.910**			
	**Θ**				**Θ**			
***pg***	0.04	0.08	0.2	0.4	0.04	0.08	0.2	0.4
0.0	**0.978**	**0.978**	**0.956**	**0.940**	**0.978**	**0.978**	**0.960**	**0.948**
0.05	**0.919**	**0.914**	**0.901**	**0.887**	**0.921**	**0.916**	0.904	0.892
0.1	**0.878**	**0.875**	**0.862**	**0.846**	0.881	0.876	0.863	0.848
0.2	**0.813**	**0.807**	**0.794**	0.774	0.812	0.809	0.797	0.780
0.3	0.752	0.752	0.736	0.719	0.755	0.749	0.737	0.717
	**1980 - 1993, *N =*** **18, *M*** **= 0.856**				**1995-2009, *N*** **= 30 , *M*** **= 0.869**			
0.0	**0.978**	**0.978**	**0.957**	**0.944**	**0.978**	**0.978**	**0.961**	**0.951**
0.05	**0.919**	**0.913**	**0.904**	**0.890**	**0.919**	**0.916**	**0.906**	**0.894**
0.1	**0.878**	**0.877**	**0.862**	0.848	**0.880**	**0.877**	0.866	0.850
0.2	0.812	0.806	0.795	0.778	0.812	0.811	0.795	0.778
0.3	0.756	0.752	0.740	0.721	0.756	0.750	0.739	0.720

Used constant values for simulations: *μ* = 2 × 10^-4^ and δ_*g*_ = 3.1. Significant MC-values in Table b) are bolded. Sample from 1920–1959 was not tested owing to small sample size.

*N* = number of samples, *M* = average M-ratio over all loci, Θ = *4Neμ*, *pg* = proportion of not single-step mutations and δ*g* = mean step size.

#### ***Effective population size***

Both *N*_*e*_ estimates with single-sample methods for the wolves prior to 1920 were very small: LD-*N*_*e*_ = 20.5 (13.5–35.2) and 13.2 (10.8–19.6) with ONeSAMP method. ONeSAMP approach provided consistently lower *N*_*e*_ estimates compared to the method based on linkage disequilibrium for other temporal groups as well (Table 3). Confidence limits of the two estimates were not overlapping for samples in 1960–1979 (76.4 and 24.3) and for the present-day reference population (99.2 and 37.2) suggesting a significant difference between them.

Temporal estimates from the historical data (~25 generations interval between the samples) suggested a *N*_*e*_ ranging from 86 to 176.4 (Table 5). Incorporation of modern wolf samples after the population recovery in 1995 extended our sampling interval with additional five generations, but did not significantly change the magnitude of the estimates of any of the used methods. Although there was an over 2-fold difference between the smallest and largest estimate of *N*_*e*_ on the museum sampling interval, confidence intervals were rather wide and overlapping, and estimates hence not significantly different.

**Table 5:** **Temporal *N***_***e ***_**estimates with 95% confidence intervals for the Finnish wolves with the modern-day reference population sample included (~30 generations) and for the historical samples only (~25 generations)**

	**Method**		
**Sampling interval**	**Moment based (1)**	**Pseudo-ML (2)**	**Coalescent Bayesian (3)**
~25 generations	86 (63–139)	153.2 (102.3 - 249.1)	176.4 (119.8 - 283.0)
~30 generations	77 (58–115)	129.8 (100.4 - 172.3)	188.3 (136–271.8)

References to methods (1) [38], (2) [39], (3) [40].

## Discussion

Genetic analyses of DNA extracted from museum specimens allowed us to directly assess the patterns of genetic variation and structure in the historical Finnish wolf population. Demographic changes due to recent anthropogenic perturbations of wild population are often so large, that the use of present-day data solely to reconstruct the population history could be misleading [22,23]. Considering the hunting statistics as a proxy for wolf abundance, the largest reduction appears to have occurred prior the 20th century (Figure 1). Although we lack a sufficient sampling of this time period, we were able to detect intriguing changes in the genetic composition of the Finnish wolf population during the last ~150 years.

Loss of genetic diversity is assumed in conjunction with strong population size decline if gene flow is insufficient to prevent local genetic drift [1]. Our results confirmed gradual genetic change (Figure 6; Additional file 5: Figure S2) and accompanying increase in genetic differentiation (Table 2; Figures 7 and 8) within the Finnish wolf population over time. The most drastic genetic changes were the disappearance of alleles (Additional file 5: Figure S2) and haplotypes (see Tables 1 and 3 for the private richenesses of haplotypes and alleles, respectively). Almost 20% of microsatellite alleles present in the historical Finnish wolf population have not been found in a comprehensive analyses of the modern population (this study; [20,41]), and only three (37.5%) mitochondrial haplotype lineages [36] out of eight found in this study have remained (Figure 4).

Compared to the Eastern European wolf populations, which were probably less affected during the historical wolf persecution than the western populations (see Background), the number of mitochondrial haplotypes was of the same order or even higher in our oldest temporal group: In the Finnish wolf samples collected prior to 1920, 5 haplotypes were found among 18 samples (Table 1), whereas Sastre et al. [10] discovered 6 haplotypes among 47 individuals in the European part of Russia and Randi et al. [42] reported 7 haplotypes among 26 individuals in the Bulgarian population. In total, 21 haplotypes were detected among the present-day European mtDNA wolf sequences together with the historical haplotypes from this study (Figure 5). One of the historical Finnish haplotypes was unique, which suggest that some variation might be lost even on a larger geographic scale. Moreover, haplotypes lost from the modern Finnish wolf population but still found elsewhere in Europe, show much wider geographic distribution than the extant haplotypes. This observation is consistent with the substantial decline in and large-scale fragmentation of the European wolf population.

Examination of the distribution pattern of historical microsatellite variation also revealed significantly larger genetic variation among the oldest wolf samples (Additional file 5: Figure S2) and a gradual temporal shift in the Finnish wolf gene pool (Figure 7). In the FCA-plot only a few wolves in the oldest group (before 1920) were within the range of the modern wolf population (1995–2009). Similar pattern of distribution was observed between the modern-day Karelian wolves and the oldest historical wolves (Additional file 7: Figure S3) indicating that some of the past variation has likely been lost in the neighbouring areas as well. The assignment analysis (Figure 8) showed a very small probability of admixture (< 8%) of the oldest Finnish temporal sample with subsequent groups referring also to notable changes in the population gene pool. Population bottleneck tests suggested early *N*_*e*_ decline (prior to 1920; Table 4) and also for historical wolves collected after 1959 (Table 1). Even though it is likely that the Finnish wolf population underwent heavy local population declines in the 1920s and 1970s [12,16,17], our results indicate that the largest genetic changes are probably of older origin, and connected to the assumed abrupt population decline in the turn of the 19th and 20th centuries (Figure 1).

Early genetic change was further supported by clustering analysis: three genetic clusters (Figure 9) among historical Finnish wolves were found, of which one was almost exclusive to oldest samples, and all of the individuals assigned to this cluster were from Northern Finland (see also Figure 7). Interestingly, in a similar historical case study from neighbouring Scandinavian wolf population [6], genetic differentiation between the northern and southern wolves was also reported. Though we cannot be certain that the historical wolves typical to Scandinavia were similar to the Finnish ones, it is possible that some historical variation typical to northern Fennoscandia has been lost. Northern Finland and Scandinavia are traditional semidomestic reindeer management areas, where tolerance of wolves is low [16,43,44]. The efficient removal of wolves from these areas not only decreases the possibility of migration towards Scandinavia today [43,44], but might have caused the detected loss of genetic variation during historical times.

Unlike in Flagstad et al. [6] – in which a ~30% decrease in heterozygosity besides a 40% reduction of allelic diversity was reported due to population decline – the amount of genetic diversity measured by means of heterozygosity did not significantly change in the Finnish wolf population during the study period (Tables 1 and 3). However, heterozygosity is relatively insensitive to the effects of short bottlenecks, and even in the most extreme case, when only a single breeding pair would survive, 75% of the genetic heterozygosity remains in the next generation [1]. Moreover, population growth after the bottleneck and especially gene flow to post-bottlenecked population from another population can effectively counteract the (further) loss of heterozygosity [45-47]. For example, Nyström et al. [27] showed that as a result of a severe demographic bottleneck in the early 20th century, the Scandinavian arctic fox population lost about 25% of its microsatellite alleles and four of seven mtDNA haplotypes, whereas the level of heterozygosity did not significantly change most probably due to gene flow from Russia. Immigration from Russia may also have been sufficient to prevent the loss of heterozygosity and local extinction, but not to completely restrain the loss of alleles and haplotypes in the Finnish wolf population. It is also possible that heterozygosity indeed decreased in the Finnish wolf population during the largest demographic bottleneck in the late 19th and early 20th century (Figure 1), but because of the very limited amount of samples of that period, we are unable to confirm this hypothesis.

Current genetic methods detecting bottlenecks from single population samples are all based on detecting deviations from expectations under mutation-drift equilibrium, and contrast two different diversity indices, of which one is more affected by genetic drift than another [48]. Regardless of the fact that the proportion of rare alleles was ~15–45% lower in samples collected before 1960 than in later samples, the heterozygosity method failed to indicate bottlenecks in the historical Finnish wolf population (Additional file 9: Figure S5). In cases with severe, long-lasting bottlenecks together with preceding large population size – which is the likely scenario for the Finnish wolves (Figure 1) – genetic bottlenecks are more likely to be correctly detected with the *M*-ratio test [49]. The observed *M*-ratios were high in all temporal groups (0.794–0.910) and well above the often used critical value of 0.68 typical to putatively stable wild populations [50]. Simulated mutation-drift expectation values for the first temporal sample (before 1920, *M* = 0.794) were much higher than this critical value in equilibrium under the most realistic evolutionary scenarios (Table 4) supporting a preceding bottleneck. It is possible that the typical *M*-ratios in some wild populations are clearly higher (this study; [28,46,51]), which highlights the importance of estimating *M*-ratio values over a varying range of parameter values when the mutation model and *N*_*e*_ are not known in detail [48].

Mitochondrial sequences have only ¼ of the *N*_*e*_ compared to autosomal markers and lack recombination. Thus they are more prone to genetic drift. Contrary to microsatellites showing a likely bottleneck in the earliest temporal period, changes in mitochondrial diversity were more gradual, and a significant signal of demographic change was detected only in temporal groups collected at or after the 1960′s (Table 1). In conclusion – a genetic bottleneck was likely in the oldest samples based on microsatellite markers, and the population gained a new equilibrium state in later generations. On the other hand, neutrality tests with mtDNA suggested continued genetic drift and loss of genetic variation more recently (from 1960′s onward).

Our linkage disequilibrium based *N*_*e*_ estimates were always larger than those given by the ONeSAMP approach (Table 3). LD-*N*_*e*_ results are likely to more correct, because former studies [20,52,53] have shown that varying sample size may lead to biased ONeSAMP estimates, and the number of wolves in our temporal groups was quite limited. The LD-based method has also its limitations. If the linkage disequilibrium between loci is a result of something else than preceding small effective size – including substructure and overlapping generations – LD-based methods may also give biased *N*_*e*_ estimates [54,55]. Because our temporal samples were collected over an extended period, there were evidently individuals from several wolf generations in each group, but the bias is likely rather similar in different temporal groups. Point estimates of *N*_*e*_ in the first temporal group (Table 3) might also have been downward biased because there were two distinctive genetic clusters with very little admixture among samples collected prior to 1920 (Figure 9).

Some assumptions of *N*_*e*_ estimation with temporal methods were likely to be violated, and thus those estimates could also be somewhat biased. Especially violating the assumptions of no immigration and non-overlapping generations could have biased our results. With overlapping generations temporal methods tend to give a large overestimate (~50%) of *N*_*e*_ e.g. for large mammals, which have low fecundity and a Type I survivorship curve [55,56]. On the other hand, this bias is likely to be greatly alleviated with the long time frame (~25/30 generations) of the temporal groups [2,56]. The bias caused by immigration, on the other hand, is likely to be large and its effect depends on the allele frequencies in the source population: If immigrants are from genetically similar source population (which is the likely scenario in our study), *N*_*e*_ is biased high when immigration reduce the drift signal [55]. In that case, depending of the rate of immigration, estimate tends to reflect more the *N*_*e*_ of the whole metapopulation and not solely the local *N*_*e *_[2,54].

Our different estimates of the *N*_*e*_ in the Finnish wolf population during the last ~150 years suggested a historical effective size ranging from 86 to 176.4 (Table 5). With typical *N*_*e*_ /*N*_*c*_ –ratios of ~0.2–0.3 for wolves [20,57] this would suggest a mean census population size of at least ~300–900 wolves. Therefore it is clear, that immigration from the Russian population has had an explicit effect on (the effective population size of) the Finnish wolf population during historical times. On the other hand, a previous study by Aspi et al. [41] suggested an even larger ancient effective size of about 590 wolves, which started to decline exponentially in the late 19th or early 20th century (see Figure 1. which supports the earlier decline). The limited amount of samples from this time period in our study (Additional file 1: Table S1 and Additional file 10: Table S5) are likely to restrict the power of our analyses, as we may detect only a fraction of the oldest historical genetic signals. Compared to our effective population size estimates from the contemporary Finnish wolf population [20], however, mean historical effective size of 86–176 is relatively high. In general the corresponding estimates from modern population are 50-85% lower (except for very strong population growth phase in 2001–2006 with very little genetic drift) and in concordance with the assumption of very recent genetic isolation from the Russian Karelian population [13,20].

## Conclusions

The Finnish wolf population has been demographically dependent on the neighbouring Russian wolf population for a long time. Active persecution of wolves in Finland lasted for over 150 years and wolves were, and still are, actively hunted in the nearby areas in Russia. Long-term anthropogenic pressure has been shown to be able to restrain gene flow between population and cause population fragmentation even in highly mobile species like wolves [58,59]. Thus an assumption of genetic homogeneity and constant positive influx from larger source population may not hold, and especially so, if the source population itself is under continuous hunting pressure.

This study provided a long-term historical perspective of the Finnish wolf population genetics. Connectivity with the much larger Russian population was shown to have retained a high amount of nuclear genetic diversity in the small Finnish population, and no significant decrease in heterozygosity was detected. However, the majority of historical mitochondrial haplotypes and a high number of autosomal alleles appear to have been lost and a specific northern wolf type has likely disappeared from the present-day gene pool. Moreover, effects of genetic drift causing changes in allele frequencies over time was evident, and point estimates of the effective population size in general is rather small and supporting population fragmentation [60]. The largest genetic differences were observed between the oldest (prior 1920) and subsequent temporal groups suggesting that the majority of the detected genetic changes were accompanied with the strong population decline at the turn of the 19th and 20th centuries. The heavy decline was also seen with microsatellite markers as lower than expected *M-*ratio for samples collected prior 1920. Without corresponding historical sampling from Russia, we are unable to confirm if similar demographic and genetic changes have occurred in the source areas as well. However, because the present-day Karelian population also differed notably from the historical Finnish samples, it is likely that the detected changes apply to a somewhat larger geographic region.

The present-day Finnish wolf population recovered via natural immigration from Russia after 1994 and the population grew rapidly until 2006, but then the population quickly declined [20]. According to the latest (2014) census estimates by the Finnish Game and Fisheries Research Institute, there are now only ~140–155 wolves in Finland and the species is listed as endangered. After the population crash, inbreeding in the population has increased significantly [20] and gene flow between Russian Karelian and Finnish populations seems to be low at present [13,20]. In order to maintain a genetically healthy and viable wolf population in the long-term, it is clear that the population should be larger and/or better connected to the Russian population. The historical Finnish wolf population was likely much larger, genetically more diverse and more uniform with the Russian population than the population today (this study; [41]). Thus, the ultimate management goal should be to restore this connection. This can probably only be achieved with substantial reduction of the anthropogenic pressure towards wolves still prevailing on both sides of the border.

## Methods

### Sampling

One hundred and fourteen historical wolf samples from 1845–1993 were collected from zoological museums in Oulu, Helsinki and Kuopio in Finland. Collection year and location were known for most of the samples (Additional file 1: Table S1), except for four samples with no exact collection year and five samples without exact collection location. The study material consisted of several types of bones (mostly teeth, some pelvic bones, vertebrae, pieces of skull bone and femurs). If available, canines were preferred for DNA extraction because of their large size, but if they were broken or missing, other teeth were used. Other types of samples included were foot pad, claws, dried pelt samples and dry blood/neural tissue obtained inside the processed teeth (Additional file 1: Table S1). Samples were sorted into four temporal groups for the analysis: samples prior to 1920 in the first (*N* = 35), 1920–1959 in the second (*N* = 10), 1960–1979 in the third (*N* = 33) and 1980–1993 in the fourth group (*N* = 36).

A random subset of contemporary Finnish wolf samples (*N* = 30, from 1995 to 2009; [20]) collected after the recovery of the population in 1995 was used as a reference in the genetic analyses. A larger dataset of modern wolves (*N* = 298) was used for illustration and comparison of the distribution of genetic variation in different temporal groups, and for comparison of the amount of private alleles between historical and present-day wolves. Collection locations of the analysed historical samples together with the corresponding temporal grouping are presented in Figure 2. Samples from the neighbouring, present-day Karelian wolf population (1995–2010, *N* = 37; for sample information see [13,20]) were used to evaluate the genetic distinctiveness of the historic wolf population.

### Sample preparation and DNA extraction from historical samples

Genetic analysis of DNA extracted from museum specimens or other old material is challenging and requires special precautions. Post mortem degradation and inhibiting substances often yield target DNA of low quality and/or quantity, and samples are prone to contamination with foreign DNA [21,61]. Low quality/quantity DNA may for example lead to erroneous base pairs in DNA sequences [34] inflating the amount of genetic variation. On the other hand, genetic variation measured by microsatellite markers may be underestimated as low quality samples are more prone to allelic drop-outs leading to erroneous estimates of homozygous genotypes. Age of the sample, sample type [21,31], preservation methods used [62,63], and storage conditions [64] may have a large effect on amplification success. To ensure the authenticity of our results, several precautions were employed throughout this study.

All work phases before polymerase chain reaction (PCR) were conducted in a laboratory where no DNA handling was previously conducted and where no physical connection to other laboratories existed. The ISO5 class laboratory is equipped with efficient filtering units and separate, positive air displacement. Special clean room clothing with a mask was used while working in the laboratory. Work phases were separated into two rooms: Sample preparation (“dirty work” e.g. cleaning, drilling and crushing of bones) was done in one room in a laminar flow hood equipped with extra suction fan and UV-light. Working equipment and work spaces were thoroughly cleaned between every sample preparation, and no more than six samples were handled simultaneously. In each extraction round two negative controls were added. The laminar hood and equipment used were UV illuminated overnight between extractions. The buffer preparation, DNA extraction and setup of PCR were done in a second room, which had a separate PCR preparation hood (PCR-Workstation, peQLab, Biotechnologie GmbH). Several blank controls were run in each PCR reaction. Clean room clothing was changed every working day and procedures were separated in a way that no walking from dust-producing bone preparation room to other room was required during the same day.

Bone sample preparation and DNA extraction followed the protocol suggested by Rohland & Hofreiter [64]. Before cutting, bones were thoroughly cleaned with HPLC-grade water moistened wipes. Outer surface of the bone (dirt containing possible inhibitors and contaminants) was removed with a Dremel® grinding tool before continuing sample removal by further drilling. Single-use grinding wheels were used for the surface cleaning and removal of the bone samples. To avoid overheating and potential DNA degradation, continuous contact of the drill with a bone was avoided [6]. After a bone sample was removed, it was crushed in a steel mortar with a heavy steel rod into fine powder inside several time folded UV-sterilized aluminium foil pocket. Approximately 0.5 g of bone powder was used for DNA extraction. In cases where less than ~0.4 g of powder was obtained, the extraction protocol was scaled and volumes adapted for smaller amounts [64]. In many cases dried blood or neural tissue was obtained inside the dental cavity. Such samples were salvaged, cut into smaller pieces with scalpel and extracted with DNeasy extraction kit (Qiagen) following the manufacturer’s protocol. The claw samples were prepared in the same way as bones but extracted in a way similar to tissue samples. However, for claw samples the amount of Proteinase K was doubled, and the final two eluation steps were done with 50 μl volume. From the dried pelt and toe pad samples, a piece of tissue (~1 cm × 1 cm) was cut with a sterile disposable scalpel and hairs were removed. The sample was then washed with 37°C HPLC-grade water and incubated for 1 hour in 55°C HPLC-grade water for further removal of contaminants and softening the tissue for cutting. After that the sample was cut into pieces with a scalpel and extracted with the DNeasy extraction kit. If DNA amplification failed with both eluates, an additional step of 100% ethanol wash was added prior to water incubation in a new extraction trial.

### Molecular methods

#### ***Mitochondrial DNA***

All historical samples were amplified for a ~450 bp long mitochondrial control region fragment using primers ClCRleft and ClCRright (Additional file 11: Table S4). Primers were designed using software Primer3 [65] for the target area (430 bp) covering most of the reported genetic variation for wolves. Samples that did not produce a satisfactory whole-length sequence with these primers were amplified for the same region with four primer pairs producing shorter, overlapping, ~140-180 bp products (Additional file 11: Table S4). We used a criterion that the whole target area of each consensus sequence should be covered at least twice. Sequences with unique or rare haplotype were amplified and sequenced up to three more times in both directions.

PCR amplifications were performed in 25 μl volume with 2.5 mM MgCl_2_, 1X buffer, 0.1 mM dNTP’s, 2.5U of polymerase enzyme (AmpliTaq GOLD®), 2.5 μM of each primer and 0.5-3 μl of DNA extract. PCR protocol included 4 min denaturation at 95°C followed by 11 cycles with 30 s at 95°C, 30 s at 65°C in the first round and thereafter decrease by 1°C for each cycle and 1 min at 72°C and 45 cycles 30 s at 95°C, 30 s at 52°C and 60 s at 72°C and a final extension at 72°C for 8 min. Negative extraction and PCR controls were run along the samples in each PCR reaction to detect possible contaminations. PCR products were purified using FastAP™ (Fermentas) –ExoI method as described in the manufacturer’s info sheet. After that they were sequenced with BigDye™ Terminator v3.1 kit (Applied Biosystems). Unincorporated dye was removed using Sephadex® (GE Healthcare) method. Sequencing was performed on an ABI 3730 DNA analyser (PerkinElmer Applied Biosystems) and gel runs visually checked with Sequencher (v. 4.7; Gene Codes corporation).

#### ***Microsatellite amplification***

Extracted historical wolf DNA was amplified with 15 microsatellite loci [66-68]; Dog Genome Project 12 of which were dinucleotide (C20.253, C09.173, CXX.225, CPH2, CPH4, CPH8, CPH12, REN169O18, AHT137, AHTH130, INRA21, AHTk211) and three tetranucleotide repeats (C2001, C2088, C2096). PCR amplification was performed in10 μl reaction mix containing 1 × AmpliTaq® 360 Buffer, 2 mM MgCl_2_, 0.2 mM each dNTP, 0.4 μM of forward and reverse amplification primer, 0.3 U AmpliTaq® 360 DNA Polymerase and 1 μl of template DNA. The forward primer of each primer pair was labelled with fluorescent dye. The PCR profile for primers C20.253, C09.173, CXX.225, CPH2, CPH4, CPH8, CPH12, C2001, C2088 and C2096 was set up with an initial denaturation phase of 5 min at 95°C followed by 11 cycles with 30 s at 95°C, 30 s at 58°C (then -0.5°C per cycle) and 1 min at 72°C and 38 cycles with 30 s at 95°C, 30 s at 50°C and 1 min at 72°C. Final extension at 72°C for 10 min concluded the reaction. Five other loci (REN169O18, AHT137, AHTH130, INRA21 and AHTk211) had first 5 min at 95°C followed by 5 cycles with 30 s at 95°C and 45 s at 65°C (then -1°C for each cycle), 11 cycles with 30 s at 95°C and 30 s at 60°C (then -1°C for each cycle) and 1 min at 72°C, 28 cycles 30 s at 95°C, 30 s at 55°C and 1 min at 72°C, and a final extension of 10 min at 72°C.

The quality of extracted DNA was first tested with two microsatellite loci (C2096 and CPH2) with relatively short products (< 110 bp) and previously known to amplify well even with degraded DNA. Samples that gave products for these loci were amplified for all 15 loci. All amplifications were repeated and results interpreted independently at least three times by two people. A heterozygote genotype was not validated unless each allele had been observed twice and a homozygote unless three amplifications were consistently homozygous. If these requirements were not met after five amplifications, half locus or missing data for a given locus was recorded. Target amplicon length affects the amplification success in samples with degraded DNA and therefore fragments < 200 bp should generally be used [21,28]. We retained all loci for amplification and subsequently evaluated whether longer amplicon lengths had higher error rates and/or poorer amplification success. The effect of sample age and sample type on amplification success was also monitored. Additionally, MICROCHECKER 2.2.3 [32] was used for all loci to test for possible genotyping errors due to stuttering or large allelic drop out and the presence of null alleles.

### Genetic data analysis

#### ***Mitochondrial sequence analyses***

##### 

**Mitochondrial variation and population bottleneck tests** Sequence variability in population was quantified as the number of polymorphic sites (*S*), the number of haplotypes (*H*), haplotype diversity (*H*_*d*_) and nucleotide diversity (π) with the software ARLEQUIN (version 3.5.1.3.; [69]). Because sample sizes varied between the temporal groups, haplotype richness and private haplotype richness were also estimated with the rarefaction method using the software HP-rare [70,71]. ARLEQUIN was also used for estimation of genetic differentiation (Φ_*ST*_) between the temporal groups based on pairwise differences. Additionally, Tajima’s *D *[72] and Fu’s *F*_*S *_[73] tests implemented in ARLEQUIN were ran with 1000 simulations to test the null hypothesis of demographic stability and possible bottlenecks. Bottleneck tests of neutral sequences under mutation-drift equilibrium are based on the expectation, that while the number of segregating sites (and especially rare ones) and distribution of haplotypes decline quickly with low *N*_*e*_, whereas nucleotide diversity is less affected [48,73].

##### 

**Phylogenetic analysis of mtDNA sequences** Temporal haplotype network for the historical mtDNA consensus sequences was constructed using the TempNet script [74] in R [75]. To examine phylogenetic relationships on a larger geographic scale, we obtained all available European wolf mtDNA control region sequences (*N* = 656) from GenBank and aligned them with our historical wolf sequences using the CLUSTAL W function [76] implemented in MEGA 5.0 [77]. Only sequences for which geographic location was available were included in our analysis. Because many published mtDNA wolf sequences are substantially shorter than our sequence (431 bp), we constructed two different phylogenies for a sequence length of 390 bp (*N* = 472) and 287 bp (*N* = 497) discarding shorter sequences. According to substitution model tests in MEGA, Kimura’s two parameter model K2P; [78] with gamma parameter of 0.05 was the best model of DNA sequence evolution for both sets. A phylogenetic tree for the haplotypes was constructed using the neighbour-joining method [79] and using the K2P model. Bootstrap values were obtained using 1000 replicates. Distinct Indian wolf lineages (*Canis indica* and *C. himalayensis*), which are suggested to be among the most ancient wolf lineages [80], were used as an outgroup.

### Microsatellite analyses

#### ***Genetic variation, HW-equilibrium and linkage disequilibrium***

The software GENETIX (version 4.05.2.; [81]) was used to compute the observed and expected heterozygosities for each temporal wolf group. Inbreeding coefficients and their 95% confidence intervals based on 1000 permutations were also estimated with the same program. FSTAT (version 2.9.3.; [82]) was used for allele number and allelic richness estimation. Private allelic richness in each temporal group was calculated with HP-rare [70,71] using the rarefaction method that compensates uneven sampling. The possible presence of alleles that are lost in modern samples (‘ghost alleles’; [28,83,84]) was examined. Genetic variability and inbreeding coefficients between groups were compared using the randomization method implemented in FSTAT by combining wolf samples collected before 1960 into the first group, and samples collected in 1960–2009 into the second one. The two-sided P-values were obtained using 1000 permutations. Individual heterozygosity (the proportion of heterozygous loci) was calculated for all individuals and Spearman’s rank correlation between the sampling year and individual heterozygosity was estimated.

Hardy-Weinberg equilibrium in each temporal group was tested with the exact test (Markov chain; 10000 dememorisations, 20 batches and 5000 iterations per batch) using GENEPOP 4.0.10 [85]. Global tests across loci and groups were performed with Fisher’s method. Pairwise genotypic linkage disequilibrium tests between all pairs of loci in each temporal group were calculated with ARLEQUIN with 1000 permutations using the EM algorithm and significance level of 0.001.

#### ***Genetic differentiation and distribution of variation***

The distribution of genetic variation within and between the groups (AMOVA) and genetic differentiation (*F*_*ST*_) between the temporal groups were estimated with the program ARLEQUIN. The significance tests for global AMOVA (weighted average over all 15 loci) results were based on 1023 permutations. Population pairwise *F*_*ST*_*-*values were calculated with the distance method [86]. Time difference in years between the median sampling years between each temporal group was calculated. The Mantel test [87] was performed for the obtained time matrix together with the corresponding *F*_*ST*_ matrix in TFPGA v1.3 [88] to see if the amount of genetic differentiation between the groups was correlated with the passage of time. Significance testing was based on 10 000 permutations, which is a realistic minimum for estimating a significance level of ~0.01 [87].

The distribution of genetic variation across historical and modern wolves were analysed with a factorial correspondence analysis (FCA; implemented in GENETIX), which generates axes that describe the maximum genetic variation among individuals and plots individuals along these axes according to their genotype. To see, if genetic variation patterns were significantly different between the temporal groups, we compared the means and variances of individual scores along the first and second FCA axis between the first and present-day samples. With a null hypothesis of equality, obtained means and variances of the two FC axes were tested with *t*-test and Levene’s test, respectively.

We conducted an assignment analysis to get further information on the differentiation of the temporal groups. We performed assignment runs for the samples using the Rannala and Mountain [89] Bayesian individual assignment method as implemented in the program GENECLASS2 [90] to estimate the likelihood that a wolf originated from a given temporal population. The marginal probability of a given individual multilocus genotype was compared to the distribution of the marginal probabilities of randomly generated multilocus genotypes (1000 replicates) using the resampling method of Paetkau et al. [91]. We estimated the mean probability of assignment for each individual in a given temporal group. Even though sampled wolves could in reality belong only to one corresponding temporal group, the means of individual assignments to different samples gives us information on the magnitude of the temporal changes in the gene pool (via the division of shared genetic variation across time).

Finally, we compared the oldest Finnish historical wolves with the present-day Russian Karelian reference population (*N* = 37; [20]) to see the extent to which historical genetic variation has been retained in this much larger source population. Pairwise genetic differentiation (*F*_*ST*_) between these population samples was calculated and the distribution of genetic variation among individuals visualized with a FCA plot.

#### ***Hidden population structure***

We used the Bayesian clustering program STRUCTURE (2.3.3; [92]) to investigate the spatial and temporal cohesion of the Finnish wolf population. Each temporal group and a random subset of 30 individuals from the modern-day population [20] were included in the analysis. We performed 10 runs at each value of the fixed parameter *K* (the number of clusters), from *K* = 1 to *K* = 15. Each run consisted of 500 000 replicates of the MCMC after a burn-in of 100 000. We used the admixture model and allowed the allele frequencies to be correlated among temporal groups (see [93]). All other parameters were set to default values. The program Harvester (v.0.6.92; [94]) was used to visualize the Structure results and implement the Evanno et al. [37] ad hoc method, which detects the uppermost level of hierarchy. Statistical significance of individual assignments (q) into the detected clusters was tested by calculating 90% confidence intervals with STRUCTURE.

#### ***Demographic changes***

We used the software BOTTLENECK (version 1.2.02; [95]) to investigate the possible severe decrease of effective population size (*N*_*e*_) preceding each study period. The test was performed for all temporal groups using the two-phase model (TPM) of microsatellite evolution and a probability of 95% for single-step mutations with variance of 12 as suggested by Piry et al. [96]. The Wilcoxon test was used to determine if significant excess of heterozygosity based on observed number of alleles (*H*_*e*_ *> H*_*eq*_*,* see [96]) was detected. This would indicate a previous population bottleneck, because rare alleles are easily lost in bottlenecks but they contribute little to overall heterozygosity [95]. As a consequence of rare allele loss, frequency classes based on allele sizes shift from the normal L-shaped distribution. Mode shift test implemented in BOTTLENECK was used to test if such distortions were present in the temporal populations.

Reduction in effective population size can also create gaps in the size distribution of alleles, which can be quantified as M-ratio, the mean ratio across all loci of the number of alleles to the allele size range [50]. Averaged M-ratios in each temporal group were obtained and compared to simulated equilibrium, pre-bottleneck population with methods described in Garza & Williamson [50]. The period 1920–1959 was not analysed due to small sample size. For simulation scenarios we used a constant mutation rate (*μ*) of 2 × 10^-4^ /locus/generation (which is a realistic estimate for canine datasets with most loci being dinucleotides; [97]) with four different effective sizes (50, 100, 250 and 500) and corresponding Θ (4*N*_*e*_μ) of 0.04, 0.08, 0.2 and 0.4. As a proportion of other than one-step mutations (*p*_*g*_) five different values were used: 0.00, 0.05, 0.10, 0.20 and 0.30 and the mean size of multi-step mutations (δ_*g*_) was set to 3.1 (see [48]). Critical value, *M*_*c*_ indicating genetic bottleneck was obtained from 10 000 simulations with the lowest 5% of data determining the limit.

#### ***Effective population size***

We used two single-sample approaches to estimate effective population sizes in each temporal group separately. First, we used a linkage disequilibrium (LD) based effective population size estimator, LD-*N*_*e *_[98]. We used a monogamous mating model and excluded alleles below frequency of 0.05 from the analysis. Second, we used another single sample *N*_*e*_ estimator, ONeSAMP [99], which is an approximate Bayesian computation method combining eight summary statistics. We used priors of 2 to 500 for *N*_*e*_:s in each temporal group. Samples collected 1920–1959 were excluded due to small sample size.

Moreover, we used three different temporal methods, which estimate *N*_*e*_ from genetic change between two or more sampling points (for reviews of *N*_*e*_ estimation, see e.g. [2,55]. Median sampling year for each temporal group was calculated and used as a time point for that sample. Sampling interval was based on estimated generation time of ~3.4 years [41], according to which temporal groups after the first time period (before 1920) were approximately 15, 22, 25, and 30 generations apart. Overlapping generations might produce severe biases in temporal estimates, which are probably reduced by longer sampling interval [54-56]. Therefore, we used two distant sample pairs, which encompass the whole historical sampling interval (~25 generations; before 1920/1980–1993), or included the present-day reference population as well (~30 generations; before 1920/1995–2009).

First we used the program TempoFS [38] to estimate *N*_*e*_: s. It is moment-based, i.e. it uses temporal allele frequency changes and *F* statistics to estimate *N*_*e*_. TempoFS runs were settled according to plan I [100] with a census size of 100 (*N*_*c*_ = 50 and 200 were also tested, but estimates were similar than for *N*_*c*_ = 100 and are thus not given). Next we ran our data sets with a maximum value of *N*_*e*_ set to 500 with MNE [101], which uses the pseudo-ML (maximum likelihood) method. Because we did not have historical source population samples from Russian Karelia, the isolation model (*m* = 0) was chosen, and maximum likelihood followed that described in Wang [39]. Finally, Bayesian maximum-likelihood method based on coalescence implemented in CoNe [40] was employed. It uses the importance sampling algorithm to estimate likelihoods for set *N*_*e*_ values. We tested the *N*_*e*_ likelihoods from 2 to 500 in steps of two with 1000 MC repeats for each value.

## Availability of the supporting data

The data set supporting the results of this article is included within the article and its additional files.

## Competing interests

The authors declare that they have no competing interests.

## Authors’ contributions

This study was a part of the PhD research of EJ supervised by JA and MR. Samples used in this study were collected by JA and EJ, and prepared in clean facilities and molecular laboratory by EJ and JH. Genetic analyses were made by EJ with the help from JA and MR except for mtDNA phylogeny part, which was done by JH. EJ wrote the manuscript with help from all co-authors. Final manuscript was approved by co-authors except MR, who passed away shortly before it was finished.

## Supplementary Material

Additional file 1: Table S1 List of museum specimens. Click here for file

Additional file 2: Table S2 Amplification success for the museum samples. Click here for file

Additional file 3: Figure S1 mtDNA haplotype tree (390 bp) for European wolves including Finnish museum haplotypes from this study. Click here for file

Additional file 4: Table S3 Locus-specific microsatellite results for temporal museum groups and modern-day reference data. Click here for file

Additional file 5: Figure S2 Temporal changes of allelic frequencies in the museum data. Click here for file

Additional file 6 **Text S1.** Statistical analyses for the distribution of genetic variation (FCA) among the temporal museum groups. Click here for file

Additional file 7: Figure S3 Distribution of genetic variation: present-day Karelian wolves vs. Finnish wolves collected before 1920. Click here for file

Additional file 8: Figure S4 Results from Structure runs for the museum samples. Click here for file

Additional file 9: Figure S5 Distributions of allele frequencies among the museum groups and contemporary reference sample. Click here for file

Additional file 10: Table S5 Data set. Click here for file

Additional file 11: Table S4 Primer sequences for mtDNA amplification. Click here for file
