# Supplementary material for: Living on the edge: reconstructing the genetic history of the Finnish wolf population
Source: BMC Evol Biol. 2014 Mar 28;14:64. doi: 10.1186/1471-2148-14-64 (PMC4033686; doi:10.1186/1471-2148-14-64)
Supplement: Additional file 1: Table S1 — List of museum specimens. [file 1471-2148-14-64-S1.pdf]

TableS1 List of museum specimens, sample material, collection year and the sampling locality. Successful amplification is indicated with (X), *NA* sample not amplified with mitochondrial primers.

**Sample number, sex**	**Time of collection**	**Sampling locality**	**Sample type**	**mtDNA amplification**	**Microsatellite genotyping**
46805, ?	19th century	(Finland)	dental bone	X	
46822, ?	19th century	(Finland)	dental bone		
400, ?	1845	Pornainen	pelt		
291, f	1854	Pornainen	neural tissue in dental cavity	X	X
578, f	1878	Mäntsälä	jaw bones	X	
577, m	1880	Valkeakoski	piece of skull		
359, m	Late 19th century	Turku	claw	*NA*	X
185, ?	Early 20th century	(Finland)	pelt	X	X
101, f	1902	Kuopio	claw	X	X
39574, ?	1904	Inari	dental bone	X	X
1346, ?	1905	Nivala	dental bone	X	
227, ?	1906	Sukeva	dental bone	X	
904, ?	1907	Kirkkonummi	pelt	X	X
39390, ?	1907	Inari	dry blood in dental cavity	X	
46763, ?	1907	Nivala	dental bone		
46764, ?	1907	Inari	dental bone	X	X
46765, ?	1907	Inari	dental bone		
46766, ?	1907	Inari	dental bone	X	
33939, ?	1907	Nivala	dental bone		
33940, ?	1907	Nivala	dental bone		
1347, ?	1908	Salmi	dental bone	X	X
1354, f	1908	Inari	dental bone		
1348, m	1909	Inari	dental bone	X	
1349, m	1909	Inari	neural tissue in dental cavity	X	X
1350, m	1909	Inari	dental bone	X	X
1351, m	1909	Inari	dental bone	X	X
1352, f	1909	Inari	dental bone	X	X
1353, ?	1909	Inari	neural tissue in dental cavity	X	X
39575, ?	1912	Inari	dry blood in dental cavity	X	X
39569, f	1912	Inari	dry blood in dental cavity	X	X
39571, ?	1912	Inari	dental bone		
3611, f	1913	Sodankylä	dental bone		
39572, ?	1914	Inari	dental bone	X	X
39576, ?	1914	Inari	dental bone	X	X
2760, ?	1918	Kauvatsa	dental bone	X	
3920, f	1934	Nivala	dental bone	x	X
4100, ?	1935	Salmi	dental bone		
100, ?	1937	Maaninka	dental bone	X	X
6393, ?	1943	Aunus	dry blood in dental cavity	X	X
4944, ?	1946	Lieksa	dental bone		X
1168, ?	1954	Utsjoki	dental bone	X	X
5355c, ?	1958	Sippola	pelt	X	X
5355a, f	1958	Sippola	dental bone	X	
5355b, f	1958	Sippola	dry blood in dental cavity	X	X
5658, f	1958	(Finland)	femur	X	X
2677, f	1962	Pielisjärvi	dry blood in dental cavity	X	
2976, m	1962	Saloinen	dental bone	X	
2552, m	1962	Suomussalmi	dry blood in dental cavity	X	
3668, m	1963	Posio	dry blood in dental cavity	X	X
3043, m	1963	Salla	vertebra	X	X
4106, f	1964	Suomussalmi	dental bone		
4138, m	1964	Suomussalmi	dental bone		
3721, m	1964	Kuhmo	scapula		X
9800, m	1972	Salla	dry blood in dental cavity	X	X

12440, m	1975	Inari	dental bone	X	
13159, m	1975	Sodankylä	dental bone	X	
25338, m	1975	Kontuselkä	piece of ear	X	X
543, m	1975	Suomussalmi	pad	X	X
12146, m	1976	Hollola	dental bone	X	X
12392, f	1976	Salla	dental bone	X	X
12165, f	1976	Salla	dental bone	X	X
12106, m	1976	Savukoski	dry blood in dental cavity	X	
12395, f	1976	Salla	dental bone	X	
547, m	1976	Nurmes	frozen tissue	X	X
13160, f	1977	Ilomantsi	dry blood in dental cavity	X	X
12391, m	1977	Tyrnävä	dry blood in dental cavity	X	
12961, m	1977	Kuhmo	dry blood in dental cavity	X	X
12442, m	1977	Temmes	dry blood in dental cavity	X	X
13165, f	1977	Ilomantsi	dental bone	X	X
13163, f	1977	Ilomantsi	dental bone	X	X
14439, f	1978	Kalajoki	dry blood in dental cavity	X	X
13307, m	1978	Tohmajärvi	dental bone	X	X
14069, f	1978	Isojoki	dental bone	X	X
14061, f	1978	Isojoki	dental bone	X	X
16878, m	1979	Sotkamo	dry blood in dental cavity	X	X
15631, m	1979	Eurajoki	dry blood in dental cavity	X	X
16879, m	1979	Siikajoki	dry blood in dental cavity	X	X
16881, f	1979	Vihanti	dental bone	X	X
20107, m	1982	Kesälahti	dental bone	X	
21222, f	1983	Inari	dental bone	X	X
20108, m	1983	(Finland)	vertebra	X	
31491, ?	1983	Kiuruvesi	pelt		
26215, f	1984	Sotkamo	dry blood in dental cavity	X	X
26471, f	1984	Porvoo	dental bone	X	X
34635, ?	1985	Hyrynsalmi	dental bone	X	X
22289, f	1986	Sotkamo	rib	X	X
22290, m	1986	Sotkamo	rib		X
23837, f	1986	Kuhmo	pelvic bone	X	X
46769,m	1986	Hattula	dental bone		
21977, m	1987	Kuhmo	pelvic bone	X	X
34471, m	1987	Kuusamo	dental bone	X	
34338, ?	1987	Kuusano	pelt		
22293, f	1988	Kuhmo	vertebra	X	
22292, f	1988	Kuhmo	pelvic bone	X	X
34334, f	1989	Salla	dry blood in dental cavity	X	X
34472, m	1989	Salla	dry blood in dental cavity	X	X
34653, f	1989	Salla	dry blood in dental cavity	X	
34106, ?	1989	Salla	pelt		
8780/4279, m	1990	Salla	pelt		
34649, f	1990	Sodankylä	dental bone	X	
34650, f	1990	Salla	dry blood in dental cavity	X	X
34652, m	1990	Sodankylä	dry blood in dental cavity	X	
34654,m	1990	Salla	dry blood in dental cavity	X	X
34105, ?	1990	Salla	pelt		
34029, ?	1991	Hollola	dental bone	X	
34636, m	1991	Salla	dry blood in dental cavity	X	
34637, f	1991	Salla	dry blood in dental cavity	X	X
34648, m	1991	Salla	dental bone	X	X
34651	1991	Sodankylä	dental bone	X	X
33955, m	1992	Salla	dry blood in dental cavity	X	X
33956, m	1992	Salla	dental bone	X	
34354, m	1993	Vaasa	dental bone		
34390, m	1993	Karjaa	dry blood in dental cavity	X	X
34434, f	1993	Anjalankoski	dental bone	X	
