# Supplementary material for: Living on the edge: reconstructing the genetic history of the Finnish wolf population
Source: BMC Evol Biol. 2014 Mar 28;14:64. doi: 10.1186/1471-2148-14-64 (PMC4033686; doi:10.1186/1471-2148-14-64)
Supplement: Additional file 2: Table S2 — Amplification success for the museum samples. [file 1471-2148-14-64-S2.pdf]

TableS2 Number of samples and amplification success for historical Finnish wolves divided into four different temporal groups. In each cell is shown the number of samples in that category and below amplification success rate for mtDNA/microsatellites.

**Sample type**	**Temporal group**									
	**Before 1920**		**1920 - 1959**		**1960 - 1979**		**1980 - 1993**			
**Dental bone**	22		6		7		13		***N* = 58**	
	0.636	0.364	0.667	0.500	0.882	0.588	0.769	0.385	**0.741**	**0.448**
**Tissue inside dental cavity**	6		2		11		11		***N* = 30**	
	1	0.833	1	1	1	0.727	1	0.727	**1**	**0.767**
**Other bones**	2		1		2		7		***N* = 12**	
	0.500	0	1	1	0.500	1	0.857	0.714	**0.750**	**0.667**
**Pelt samples**	3		1		1		5		***N* = 10**	
	0.670	0.670	1	1	1	1	0	0	**0.400**	**0.400**
**Other**	2 *		0		2 **		0		***N* = 4**	
	1	1			1	1			**1**	**1**
Number of samples	***N* = 35**		***N* = 10**		***N* = 33**		***N* = 36**		***N* = 114**	
Prop. amplified mtDNA/microsat.	**0.706**	**0.486**	**0.800**	**0.700**	**0.909**	**0.697**	**0.750**	**0.500**	**0.788**	**0.579**

* two claw samples; mtDNA amplification done for one sample, ** foot pad and fresh frozen tissue samples
