# Supplementary material for: Living on the edge: reconstructing the genetic history of the Finnish wolf population
Source: BMC Evol Biol. 2014 Mar 28;14:64. doi: 10.1186/1471-2148-14-64 (PMC4033686; doi:10.1186/1471-2148-14-64)

FigureS1 European-wide wolf mtDNA haplotype tree based on 472 sequences of 390 bp in length collected from the GenBank, together with the haplotypes found in the historical Finnish wolf population (**FIN**; $N = 8$) and those existing today (**FIN**; $N = 3$).

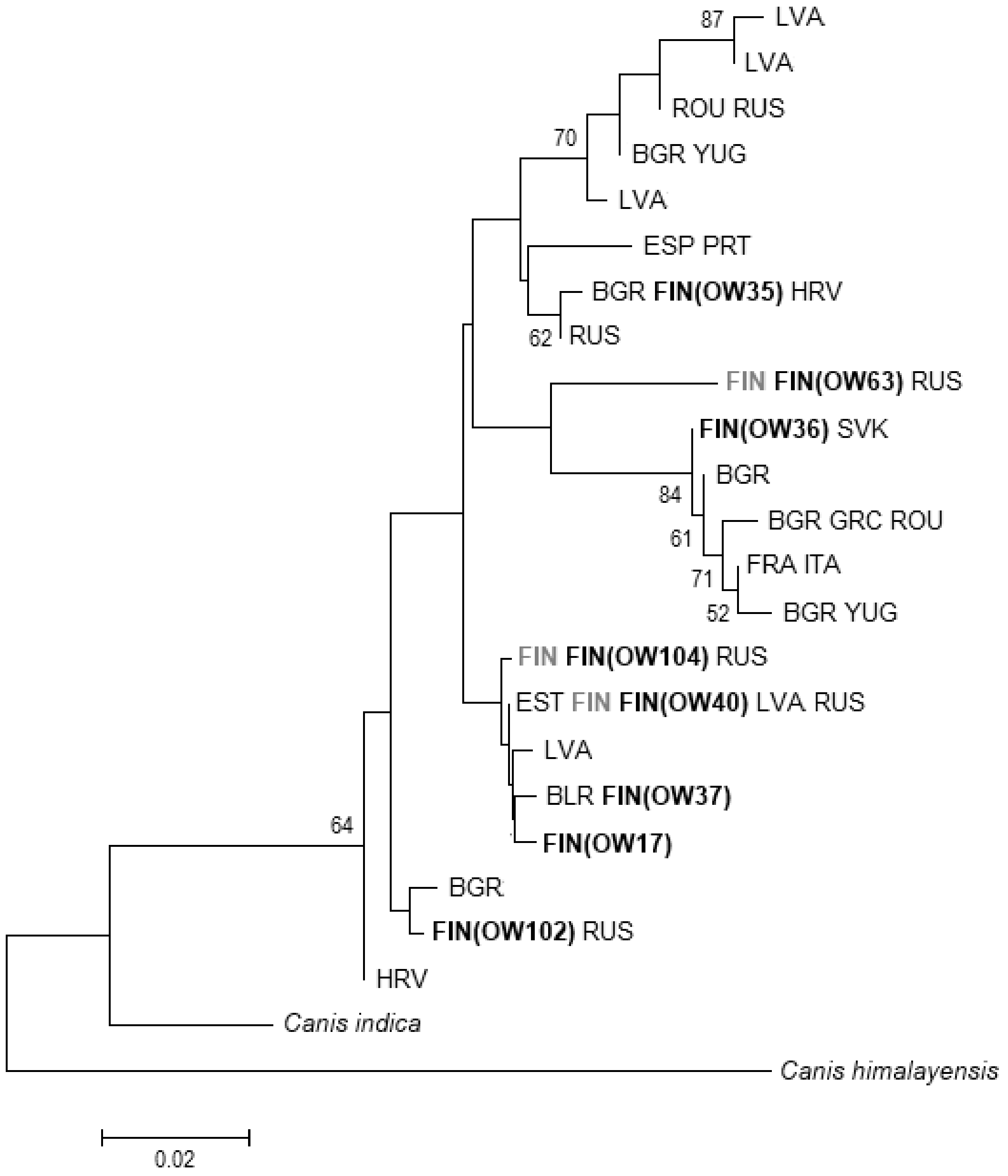

Supplement: Additional file 3: Figure S1 — mtDNA haplotype tree (390 bp) for European wolves including Finnish museum haplotypes from this study. [file 1471-2148-14-64-S3.pdf]
