# Supplementary material for: Living on the edge: reconstructing the genetic history of the Finnish wolf population
Source: BMC Evol Biol. 2014 Mar 28;14:64. doi: 10.1186/1471-2148-14-64 (PMC4033686; doi:10.1186/1471-2148-14-64)
Supplement: Additional file 4: Table S3 — Locus-specific microsatellite results for temporal museum groups and modern-day reference data. [file 1471-2148-14-64-S4.pdf]

TableS3 Locus-specific results for temporal museum groups and modern-day reference data showing expected (H_e) and observed (H_o) heterozygosities, number of alleles (A), allelic richnesses (A_R) and inbreeding coefficients (F_{IS}).

	Samples collected 1854-1919 N = 12					Samples collected 1920-1959 N = 6					Samples collected 1960-1979 N = 22				
**Locus**	H_e	H_o	A	A_R	F_{IS}	H_e	H_o	A	A_R	F_{IS}	H_e	H_o	A	A_R	F_{IS}
C20.253	0.694	0.546	6	4.10	0.259	0.722	0.833	5	4.22	-0.064	0.700	0.571	5	3.55	0.207
C2001	0.517	0.500	4	3.01	0.077	0.719	0.250	4	4.00	0.727	0.725	0.727	6	3.71	0.020
C2088	0.760	0.583	5	4.01	0.274	0.700	0.400	4	3.78	0.515	0.773	0.810	5	4.06	-0.023
C2096	0.611	0.583	3	2.81	0.089	0.708	0.667	4	3.64	0.149	0.686	0.818	5	3.39	-0.170
C09.173	0.663	0.833	4	3.45	-0.215	0.722	0.833	5	4.22	-0.064	0.587	0.790	4	2.72	-0.320
CXX.225	0.715	0.727	4	3.53	0.030	0.580	0.800	3	2.80	-0.280	0.608	0.762	4	3.18	-0.231
CPH2	0.754	0.583	6	4.22	0.267	0.778	0.833	5	4.52	0.020	0.707	0.773	6	3.73	-0.070
CPH4	0.722	0.500	5	3.90	0.347	0.736	0.667	5	4.24	0.184	0.693	0.857	5	3.67	-0.214
CPH8	0.735	0.778	5	4.08	0.000	0.594	0.500	3	3.00	0.294	0.676	0.563	4	3.15	0.199
CPH12	0.265	0.300	3	2.05	-0.080	0.780	1.000	5	4.73	-0.176	0.446	0.500	5	2.69	-0.095
REN169O18	0.750	0.875	6	4.36	-0.101	0.750	0.500	6	4.66	0.412	0.790	0.667	7	4.48	0.184
AHT137	0.785	0.636	6	4.40	0.235	0.736	0.833	6	4.65	-0.042	0.785	0.905	8	4.39	-0.129
AHTH130	0.747	0.583	7	4.16	0.260	0.740	1.000	5	4.56	-0.250	0.726	0.727	7	4.09	0.022
INRA21	0.653	0.583	5	3.74	0.149	0.792	1.000	6	5.06	-0.176	0.720	0.714	6	3.69	0.032
AHTk211	0.660	0.750	6	3.91	-0.094	0.760	1.000	5	4.58	-0.212	0.664	0.750	5	3.48	-0.105
**Mean**	**0.669**	**0.624**	**5.00**	**3.72**	**0.113**	**0.721**	**0.741**	**4.73**	**4.18**	**0.079**	**0.686**	**0.729**	**5.47**	**3.60**	**-0.038**

	Samples collected 1980-1993 N = 18					Reference sample 1995-2009 N = 30				
**Locus**	H_e	H_o	A	A_R	F_{IS}	H_e	H_o	A	A_R	F_{IS}
C20.253	0.758	0.667	6	4.18	0.148	0.758	0.759	6	3.99	0.017
C2001	0.523	0.563	5	2.94	-0.042	0.633	0.667	4	3.08	-0.037
C2088	0.789	0.889	6	4.31	-0.099	0.692	0.655	7	3.61	0.071
C2096	0.707	0.611	5	3.58	0.163	0.671	0.679	5	3.33	0.007

C09.173	0.762	0.778	6	4.16	0.008	0.668	0.767	7	3.66	-0.131
CXX.225	0.678	0.563	4	3.15	0.201	0.643	0.767	3	2.85	-0.176
CPH2	0.749	0.778	6	4.09	-0.011	0.722	0.690	6	3.62	0.062
CPH4	0.752	0.778	5	3.89	-0.006	0.740	0.700	5	3.94	0.071
CPH8	0.675	0.500	4	3.44	0.308	0.630	0.643	6	3.55	-0.002
CPH12	0.293	0.133	4	2.15	0.569	0.662	0.586	6	3.29	0.132
REN169O18	0.740	0.688	7	4.26	0.103	0.763	0.857	7	4.18	-0.106
AHT137	0.855	0.706	9	5.37	0.203	0.832	0.923	9	5.00	-0.090
AHTH130	0.640	0.529	7	3.71	0.202	0.720	0.636	8	4.06	0.139
INRA21	0.704	0.647	6	3.72	0.111	0.753	0.767	5	3.93	-0.002
AHTk211	0.517	0.500	3	2.58	0.061	0.574	0.586	5	2.93	-0.004
**Mean**	**0.676**	**0.622**	**5.53**	**3.70**	**0.112**	**0.697**	**0.712**	**5.93**	**3.67**	**-0.003**
