# Supplementary material for: Living on the edge: reconstructing the genetic history of the Finnish wolf population
Source: BMC Evol Biol. 2014 Mar 28;14:64. doi: 10.1186/1471-2148-14-64 (PMC4033686; doi:10.1186/1471-2148-14-64)

FigureS2

Allele frequency changes in the Finnish wolf population

* alleles showing large shifts
\# allele unique to one time period
\+ allele not found in modern-day samples

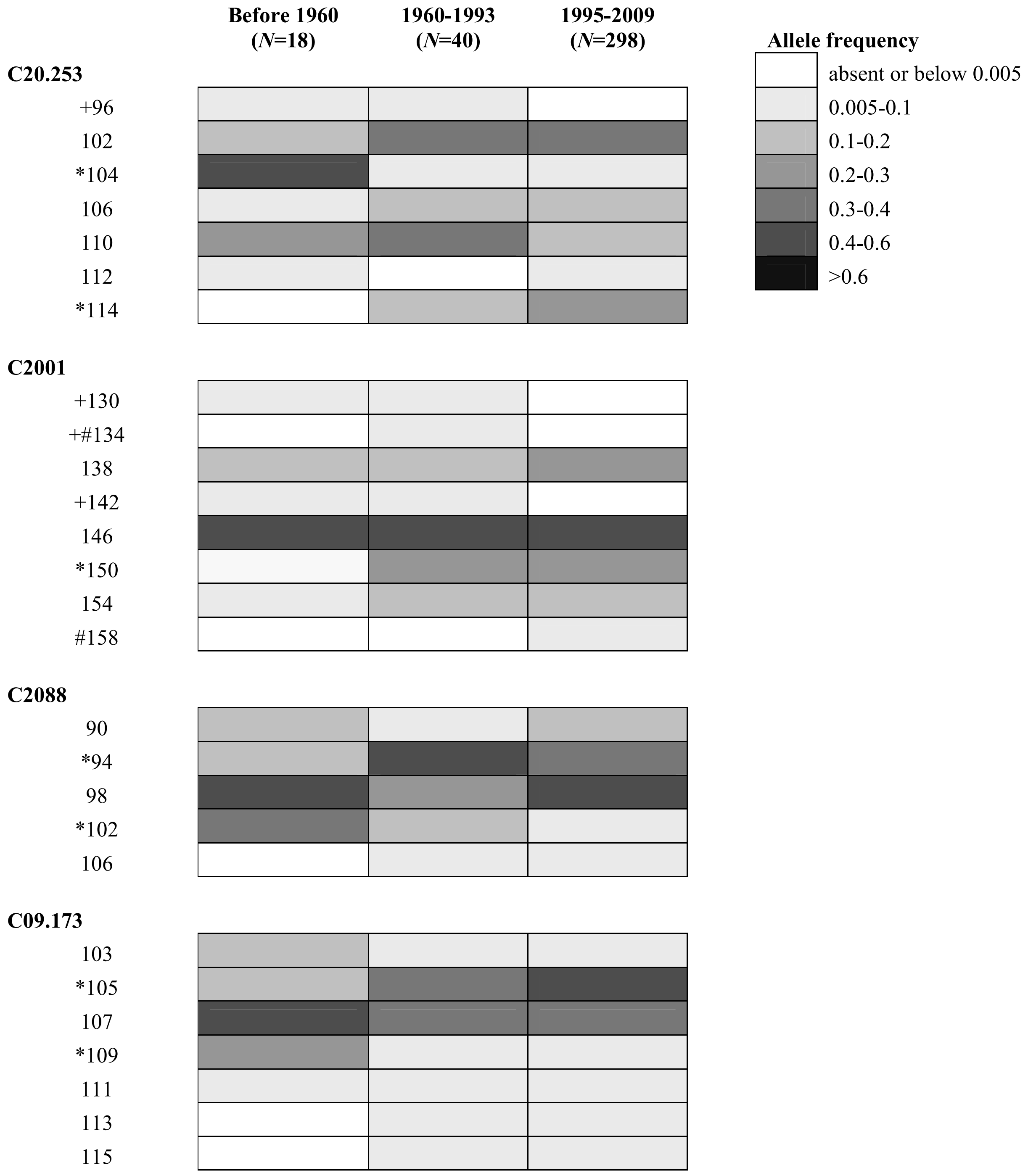

CXX.225

+#159			
161			
163			
165			
+#167			

CPH2

92			
94			
96			
*98			
100			
+#102			
104			
+#*106			
+110			

CPH4

+#*134			
138			
140			
142			
+#144			
146			
147			

CPH8

+#*196			
#200			
204			
*206			
*208			
210			
*212			
214			

CPH12

+#191			
+193			
195			
*197			

199			
203			
205			
#207			

REN169018

154			
+#156			
158			
*160			
162			
164			
166			
168			
170			

AHT137

129			
*131			
+#133			
135			
137			
141			
143			
145			
147			
149			
+#151			

AHTH130

111			
#113			
115			
117			
+119			
121			
*123			
*125			
127			
129			
131			

INRA21

+*91
+93
95
*97
99
101

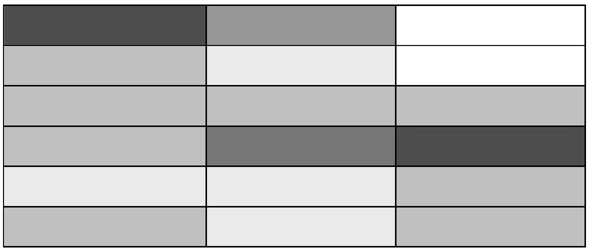

AHTk211

+#84
86
88
90
*92
94

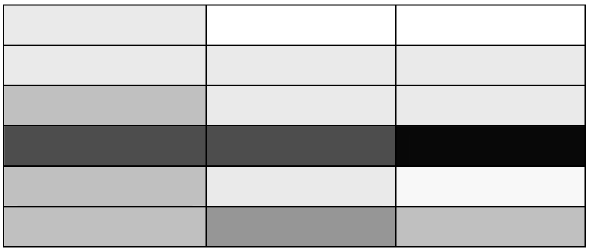

Supplement: Additional file 5: Figure S2 — Temporal changes of allelic frequencies in the museum data. [file 1471-2148-14-64-S5.pdf]
