# Supplementary material for: Living on the edge: reconstructing the genetic history of the Finnish wolf population
Source: BMC Evol Biol. 2014 Mar 28;14:64. doi: 10.1186/1471-2148-14-64 (PMC4033686; doi:10.1186/1471-2148-14-64)
Supplement: Additional file 6 — Text S1. Statistical analyses for the distribution of genetic variation (FCA) among the temporal museum groups. [file 1471-2148-14-64-S6.pdf]

TextS1

Statistical tests for the distribution of genetic variation (FCA plot) between the oldest museum samples (prior 1920) and modern-day Finnish wolf samples (1995–2009).

Variances between the groups are divergent: Levene's test for homogeneity of variances for FCA1: $W = 10.028$, $df1 = 4$, $df2 = 351$, $P = 0$, and for FCA2: $W = 3.747$, $df1 = 4$, $df2 = 351$, $P = 0.005$.

Means are divergent and the standard deviations larger in the oldest samples: The mean score of the oldest temporal sample along the first FCA-axis was 1.418 (SD = 0.671) and for the second (FCA2), 0.635 (SD = 0.619). Among the modern-day samples corresponding values were -0.117 (SD = 0.284) for FCA1 and -0.010 (SD = 0.375) for FCA2 (t-test for FCA1: $t = 17.029$, $df = 308$, $P = 0$, and for FCA2: $t = 5.676$, $df = 308$, $P = 0$).

Levene's test for variances, FCA1: $F = 30.318$, $P = 0$ and for FCA2: $F = 5.877$, $P = 0.016$.
