# Supplementary material for: Living on the edge: reconstructing the genetic history of the Finnish wolf population
Source: BMC Evol Biol. 2014 Mar 28;14:64. doi: 10.1186/1471-2148-14-64 (PMC4033686; doi:10.1186/1471-2148-14-64)

FigureS3 Distribution of genetic variation in the oldest temporal (prior to 1920) Finnish wolf group and present-day Karelian wolf samples. Historical Finnish wolves forming a distinctive northern cluster in Structure analysis are circled.

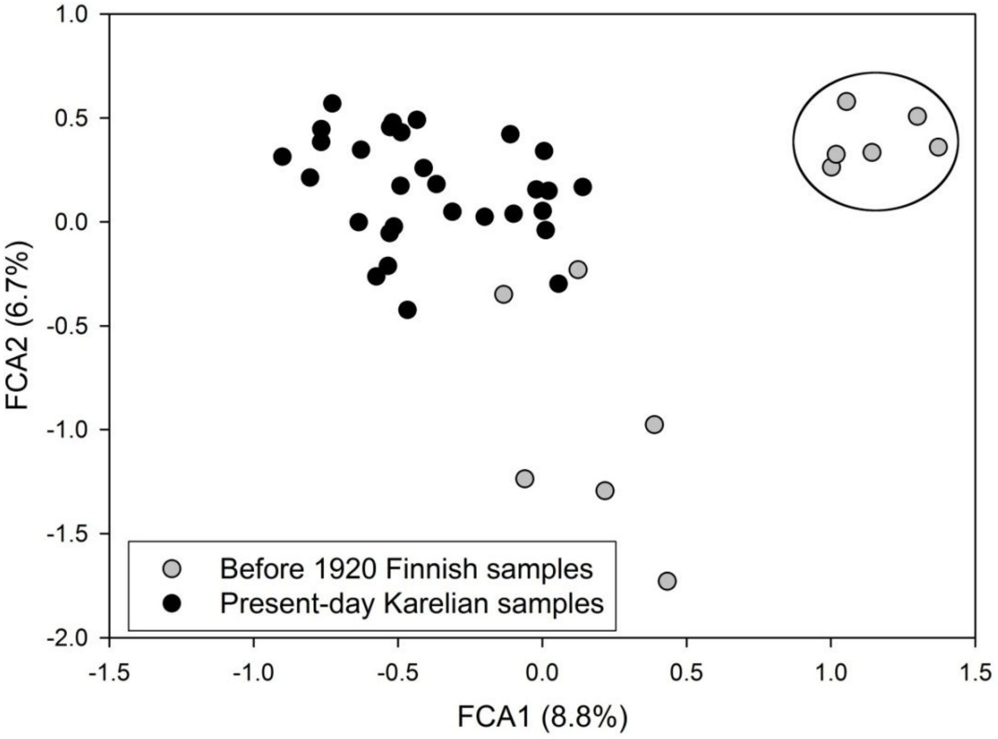

Supplement: Additional file 7: Figure S3 — Distribution of genetic variation: present-day Karelian wolves vs. Finnish wolves collected before 1920. [file 1471-2148-14-64-S7.pdf]
