# Supplementary material for: Living on the edge: reconstructing the genetic history of the Finnish wolf population
Source: BMC Evol Biol. 2014 Mar 28;14:64. doi: 10.1186/1471-2148-14-64 (PMC4033686; doi:10.1186/1471-2148-14-64)

FigureS4 Combined Structure results from 10 independent runs for each *K* value of 1-15 for historical Finnish wolves. Upper figure shows the mean (±SD) of estimated probabilities [*LnP*(*K*)] and figure below the Δ*K* values as a function of *K* where the modal value of the distribution is considered as the highest level of genetic hierarchy.

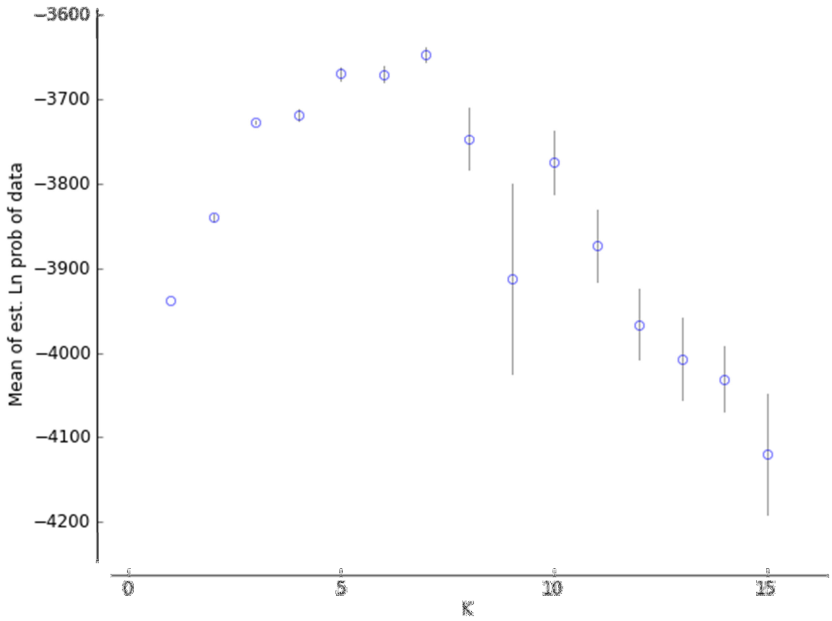

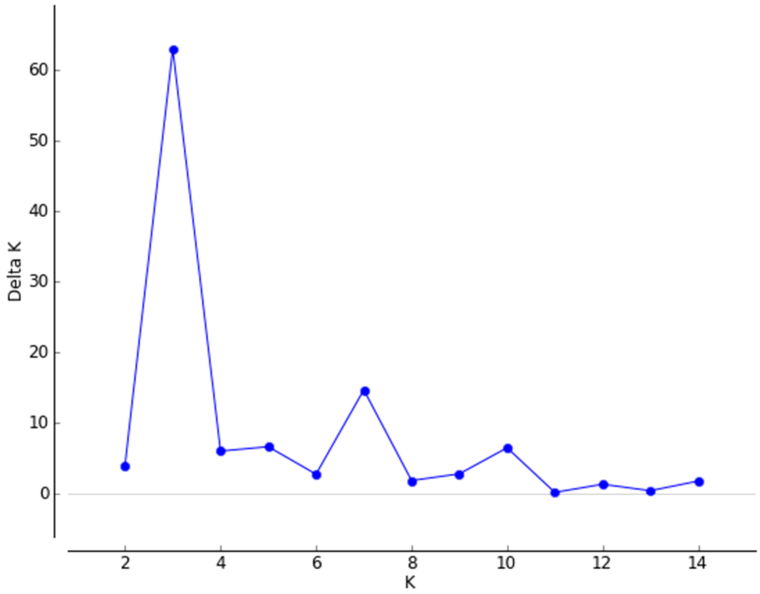

Supplement: Additional file 8: Figure S4 — Results from Structure runs for the museum samples. [file 1471-2148-14-64-S8.pdf]
