# Supplementary material for: Living on the edge: reconstructing the genetic history of the Finnish wolf population
Source: BMC Evol Biol. 2014 Mar 28;14:64. doi: 10.1186/1471-2148-14-64 (PMC4033686; doi:10.1186/1471-2148-14-64)

FigureS5 Allele frequency distributions of the microsatellite loci in the temporal groups and among the modern-day wolves.

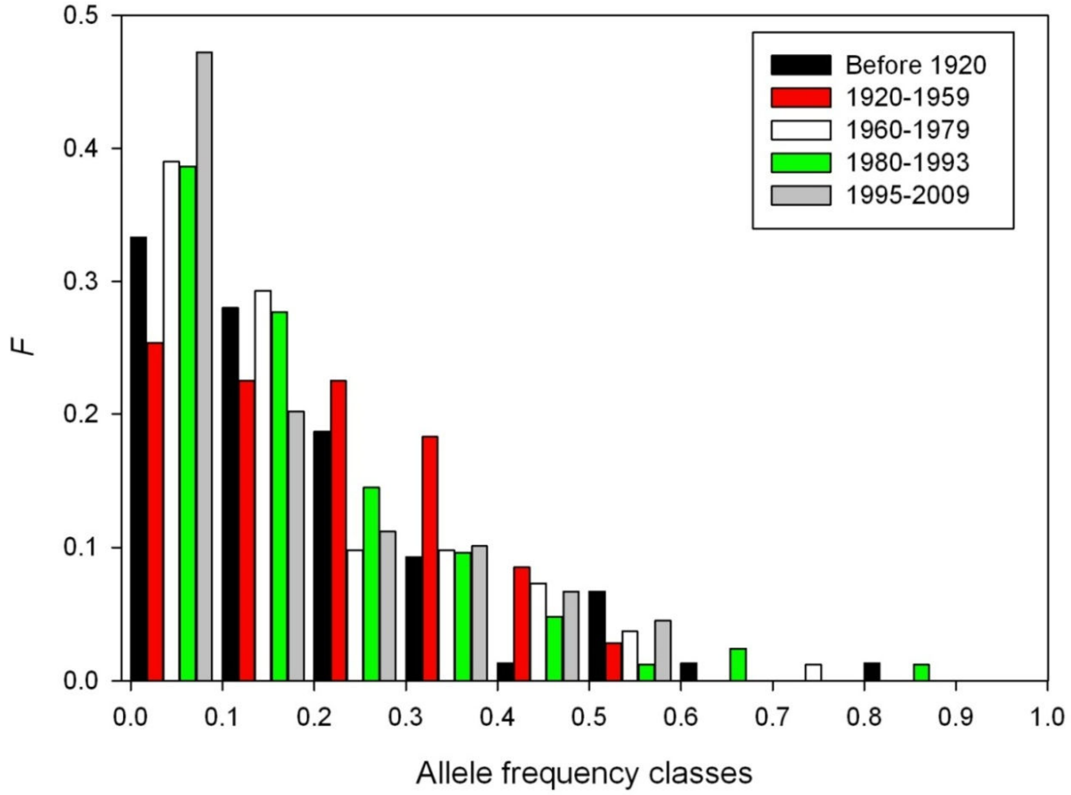

Supplement: Additional file 9: Figure S5 — Distributions of allele frequencies among the museum groups and contemporary reference sample. [file 1471-2148-14-64-S9.pdf]
