# Supplementary material for: Living on the edge: reconstructing the genetic history of the Finnish wolf population
Source: BMC Evol Biol. 2014 Mar 28;14:64. doi: 10.1186/1471-2148-14-64 (PMC4033686; doi:10.1186/1471-2148-14-64)
Supplement: Additional file 11: Table S4 — Primer sequences for mtDNA amplification. [file 1471-2148-14-64-S11.pdf]

TableS4

Table of primers used for mtDNA control region amplification. ClCR left and ClCR right amplify the whole target sequence. Number in the name of other primers tells the starting base.

Primer	Sequence (5'-3')
ClCR left	AGCACCCAAAGCTGAGATTCTTCT
Cl144R	TATGAGATTGAGTTAATATGTCCTATG
Cl77F	TGCCCCATGCATATAAGCATGTAC
Cl252R	GAAGAGGGACATTACGAGCAAGG
Cl172F	AATGCATATCACTTAGTCCAATAAG
Cl326R	GCCCTGAGGTAAGAACCAGATGC
Cl276F	GGGGGTTACTATCATGAAACTATAC
ClCR right	CAGTTATGTGTGATCATGGGCTGA
